# Plasma C5a and serum C5aR levels in patients with chronic spontaneous urticaria: A single-center case-control study

**DOI:** 10.1371/journal.pone.0351329

**Published:** 2026-06-26

**Authors:** Thao T. Pham, Minh N. Vu, My H. Le, Doanh H. Le, Thuong V. Nguyen, Phuong T.M. Pham

**Affiliations:** 1 Department of Dermatology, Hanoi Medical University, Hanoi, Vietnam; 2 National Hospital of Dermatology and Venereology, Hanoi, Vietnam; Sungkyunkwan University - Suwon Campus: Sungkyunkwan University - Natural Sciences Campus, KOREA, REPUBLIC OF

## Abstract

**Background:**

C5a and its receptor, C5aR, are involved in the degranulation of mast cells and basophils, leading to the release of proinflammatory cytokines. However, to date, their roles in the pathogenesis of chronic spontaneous urticaria (CSU) remain incompletely understood.

**Objective:**

To investigate the association of plasma C5a and serum C5aR with disease severity in Vietnamese patients with CSU.

**Methods:**

A case-control study was conducted involving 146 patients with CSU and 30 healthy adults matched by age and sex (the matching ratio ~ 5:1), at the Urticaria clinic, National Hospital of Dermatology and Venereology, Vietnam. Plasma C5a and serum C5aR levels were measured using the ELISA technique, and disease severity was assessed using the Urticaria Activity Score over 7 days (UAS7). The associations between C5a/C5aR with disease severity and several patient’s characteristics were evaluated using Spearman’s correlation and logistic regression.

**Results:**

Plasma C5a levels were significantly increased in CSU patients compared to healthy controls (p < 0.001), whereas serum C5aR levels in CSU patients were significantly reduced (p < 0.001). A significant positive correlation was found between disease severity based on UAS7 scores and plasma C5a levels (Spearman’s rho = 0.63, p < 0.001), but not for serum C5aR levels (Spearman’s rho = 0.05, p = 0.527). A C5a level of 4335 pg/mL was defined as the optimal cutoff points for identifying severe CSU with an AUC of 0.86 (95% CI: 0.80–0.92, p < 0.001), sensitivity of 64%, and specificity of 100%. Several factors were found to be associated with C5a and C5aR levels, such as prothrombin time and anti-TPO antibodies.

**Conclusions:**

CSU patients demonstrate elevated plasma C5a and reduced serum C5aR levels compared to healthy controls. While plasma C5a may serve as a potential biomarker for indicating CSU severity, C5aR levels show no significant association with the disease severity.

## Introduction

Chronic spontaneous urticaria (CSU) is defined by the recurrent appearance of wheals and/or angioedema persisting for more than six weeks without identifiable external triggers [1,2]. Affecting approximately 0.5–1% of the general population, the disease poses a substantial burden on patients’ quality of life [2,3]. Cutaneous mast cells are proved to play a central role and are primarily responsible for the pathogenesis of CSU [4]. Among the various mechanisms of mast cell activation, the IgE-dependent mechanism via the high-affinity IgE receptor (FcɛRI) has been well-established. This receptor is closely implicated in the pathogenesis of type I autoimmune urticaria characterized by the presence of IgE autoantibodies directed against autoantigens, and type IIb autoimmune urticaria, in which IgG autoantibodies target either IgE or the FcɛRI located on mast cell surface [5–7]. Emerging evidence has revealed that mast cell activation is far more complex than previously understood. Beyond FcɛRI-mediated signaling, several other receptors have also been proved to contribute to mast cell activation, including the Mas-related G protein-coupled receptor X2 (MRGPRX2), the complement component 5a receptor (C5aR), and protease-activated receptors 1 and 2 (PAR1 and PAR2) [8,9].

Activation of the coagulation system, particularly the extrinsic coagulation pathway, has been increasingly recognized in the pathophysiology of CSU [10–14]. Recent investigations have demonstrated a link between the extrinsic coagulation cascade and the complement system. Specifically, activated coagulation factors can induce cleavage of complement component 5 (C5), resulting in the generation of C5a. This potent anaphylatoxin subsequently activates mast cells and basophils via C5aR, promoting their degranulation and amplifying inflammatory responses [13,15,16]. Elevated circulating levels of C5a have been consistently reported patients with CSU compared to healthy people [12,17,18]. Relevant findings have also identified C5aR expression on the surface of various blood cells, including eosinophils, basophils, and neutrophils, as well as on tissue-resident cells such as cutaneous mast cells, sensory neurons, respiratory epithelial cells, and synovial epithelial cells, among other cell types [19–22]. Notably, soluble forms of C5aR have been detected not only in tissue samples but also in the serum of patients, suggesting systemic involvement and potential utility as a biomarker in relevant diseases [23]. Our study aimed to investigate the plasma C5a and serum C5aR level in patients with CSU and to examine their associations with disease severity as well as some clinical and laboratory features.

## Materials and methodss

### Study design and study population

A case-control study was conducted between May 2024 and December 2024 at the Urticaria Clinic, National Hospital of Dermatology and Venereology in Hanoi, Vietnam. Due to the limited number of healthy controls, CSU patients were grouped in sets of five with the closest age and each group was matched to one healthy donor of similar age. The diagnosis of CSU was established in accordance with the EAACI/GA^2^ LEN/EuroGuiderm/APAACI 2022 guidelines. Patients diagnosed or suspected of having chronic inducible urticaria (CIndU) were excluded from the study. Peripheral blood samples were collected from eligible CSU patients and healthy controls following discontinuation periods corresponding to at least five drug half-lives, in particular five days of second-generation H1 antihistamines (sgAH1), one month for immunosuppressants (systemic corticosteroids, cyclosporine A, methotrexate, etc.), and one week for NSAIDs or antibiotics. To eliminate potential confounding factors, those who had been previously diagnosed with diseases possibly influencing C5a/C5aR level, such as bronchial asthma, autoimmune hemolytic anemia, rheumatoid arthritis, and acute or chronic infections, were excluded from our study.

### Data collection and biochemical testing

Clinical data of CSU patients was obtained through medical records and was systemically taken by board-certified dermatologists trained in standardized examination procedures. The presence of CindU was determined based on medical history and a series of provocation tests, including the Frick test for dermographism, the bicycle ergometer test for cholinergic urticaria, and either the Temptest test or the ice cube test for cold urticaria. Collected clinical parameters included age, gender, disease duration, personal medical history of autoimmune conditions, family history of CSU, wheal status, angioedema, and comorbidities. Laboratory testing encompassing complete peripheral blood count (CBC), erythrocyte sedimentation rate (ERS), C-reactive protein (CRP), plasma D-dimer level, prothrombin time (PT), activated partial thromboplastin time (APTT), fibrinogen, autologous serum skin test (ASST), antinuclear antibodies (ANA), total serum IgE level, and IgG anti-thyroid peroxidase (IgG anti-TPO), were undertaken at the Department of Laboratory Medicine, which is ISO-qualified.

Plasma samples were obtained from both CSU patients and healthy donors for the quantification of C5a, while serum samples were collected for the measurement of C5aR. After collection, samples were stored at −80°C for a maximum of three months for plasma and up to six months for serum. Quantitative analyses of C5a and C5aR were performed using two distinct commercial enzyme-linked immunosorbent assay (ELISA) kits (My BioSource, Inc, San Diego, CA, USA) for each one, in accordance with the manufacturer’s instructions. All C5a and C5aR measurements were performed in duplicate, in accordance with the instructions. The accuracy of the assay was evaluated by calculating intra-assay coefficients of variation, which were 4.2% (range: 1.1% − 6.7%) for C5a and 3.5% (range: 0.8% − 6.1%). Inter-assay coefficients of variation were not determined, as the manufacturer’s protocol does not recommend or require this assessment for the kit used. All ELISA assays were conducted at the Department of Laboratory Medicine, National Hospital of Dermatology and Venereology, Hanoi, Vietnam.

The severity of CSU patients was assessed using the Urticaria Activity Score over seven days (UAS7). Patients were instructed to record daily scores for pruritus and wheal scores, each rated on a scale from 0 to 3. The UAS7 score was calculated by summing the daily scores over a consecutive seven-day period. Patients with a UAS7 score of 28 or higher were classified as severe CSU, while those with a UAS7 score below 28 were classified as non-severe CSU.

### Statistical analysis

For continuous variables, descriptive statistics were reported as means and standard deviations (SD) for data exhibiting normally distribution, and as medians and interquartile ranges (IQR) [first quartile-third quartile] for non-normally distributed data. Comparisons between cases and controls were performed using the t-test for normally distributed variables and the Mann-Whitney U test for non-normally distributed variables appropriately, with normality verified using the Shapiro-Wilk test. For categorical variables, number of cases and percentages were presented, and comparisons between groups were conducted using the Chi-squared/Fisher’s exact test appropriately.

Differences in C5a and C5aR levels were analyzed between the case and control groups, between disease severity subgroups, and between each subgroup and the control group using the Mann-Whitney U test. Correlations between C5a /C5aR levels and disease severity, as measured by UAS7, were evaluated using Spearman’s rank correlation coefficient. Logistic regression was performed to examine the associations between C5a /C5aR levels and categorical disease severity, adjusting for potential confounders including sex, age, disease duration, angioedema, elevated CRP, elevated IgE, eosinopenia, elevated IgG anti-TPO, and D-dimer levels. Receiver Operating Characteristic (ROC) curve analysis was used to determine the optimal cut-off values of C5a and C5aR for classifying disease severity. Sensitivity and specificity for predicting disease severity using the defined cut-off values for C5a and C5aR were calculated, with 95% confidence intervals (CI) determined using the exact Clopper-Pearson approach. Possible factors associated with C5a and C5aR levels in CSU patients were examined using the Mann-Whitney U test and Spearman’s correlation. To reduce the risk of false positives due to multiple testing, *P* values were adjusted using the Benjamini–Hochberg procedure. *P* value <0.05 was considered as statistical significance. All the analyses were performed using SAS software, version 9.4 (SAS Institute Inc., Cary, NC, USA).

### Ethical approval

The study received Institutional Review Board approval at Hanoi Medical University (HMUIRB#1326) and was conducted in adherence to the principles expressed in the Declaration of Helsinki. Written informed consents were obtained from all participants and healthy donors. This study followed the Strengthening the Reporting of Observational studies in Epidemiology (STROBE) reporting guideline.

## Results

### Baseline characteristics of the study cohorts

The study involved 146 eligible CSU patients and 30 healthy controls matched for age and sex. Patients’ and controls’ characteristics are presented in Table 1. Out of 146 CSU patients, 73 were classified as severe CSU and 73 as non-severe CSU based on UAS7. The D-dimer levels, elevated CRP ratios, and age were significantly higher in the severe CSU compared to the non-severe CSU. However, no significant differences were observed between the non-severe versus severe group for several factors, including sex, disease duration, family history, eosinopenia, IgE, ESR, and CRP levels, etc. There were also no discrepancies in the identification of autoantibodies, such as ANA, anti-TPO, and ASST.

**Table 1:** Demographic and disease-related characteristics of CSU patients and controls.

Characteristics	CSU patients			Controls (n = 30)	p (CSU vs. Controls)	p (non-severe vs. severe)
	Non-severe (n = 73)	Severe (n = 73)	Total (n = 146)			
Female	42 (57.53)	50 (68.49)	92 (63.01)	18 (60.0)	0.756^a^	0.170^a^
Age of study entry, median [IQR], years	28 [19-38]	35 [28, 44]	33 [22-42]	32 [27–38]	0.873^b^	**<0.001** ^b^
Age of onset, median [IQR], years	28 [18, 37]	35 [27, 43]	33 [21, 41]	NA	NA	**<0.001** ^b^
Disease duration, median [IQR]	16 [12-30]	13 [9–24]	15 [10, 25]	NA	NA	0.115^b^
Family history of CSU, n (%)	7 (9.6)	7 (9.6)	14 (9.6)	NA	NA	1.000^a^
Angioedema, n (%)	27 (37.0)	27 (37.0)	54 (37.0)	NA	NA	1.000^a^
UAS7, median [IQR]	14 [12–19]	35 [28–35]	27 [14-35]	NA	NA	**<0.001** ^a^
Eosinopenia (<0,05 x 10^9^/L)	9 (12.3)	11 (15.1)	20 (13.7)	NA	NA	0.630^b^
Elevated IgE, (>100 IU/mL)	53 (72.6)	52 (71.2)	105 (71.9)	NA	NA	0.854^b^
Elevated ESR	5 (6.8)	7 (9.6)	17 (11.6)	NA	NA	0.547^b^
Elevated CRP (> 5 mg/L)	3 (4.1)	14 (19.2)	17 (11.6)	NA	NA	**<0.001** ^b^
ANA (+)	8 (11.0)	10 (13.7)	18 (12.3)	NA	NA	0.615^b^
Elevated IgG anti-TPO, (34 kU/L)	2 (2.7)	5 (6.8)	7 (4.8)	NA	NA	0.442^c^
ASST (+)	48 (65.7)	49 (67.1)	97 (66.4)	NA	NA	0.861^b^
D-Dimer, median [IQR]	365 [270-692]	892 [452-2199]	546.5 [291- 1353]	NA	NA	**<0.001** ^a^

Abbreviations: IQR interquartile range, CSU chronic spontaneous urticaria, UAS7 Urticaria Activity Score over 7 days, IgE immunoglobulin E, ESR erythrocyte sedimentation rate, ANA antinuclear antibodies, CRP C-reactive protein, IgG immunoglobulin G, TPO Thyroid Peroxidase, ASST autologous serum skin test, NA not applicable.

Values are No. (%) unless otherwise noted.

^a^Mann-Whitney U test.

^b^Chi-square test.

^c^Fisher’s exact.

### C5a and C5aR levels

**Fig 1:**
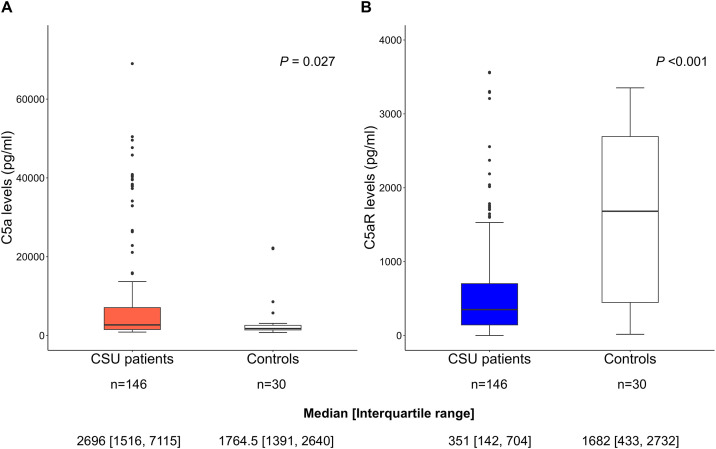
Levels of plasma C5a (A) and serum C5aR (B) among CSU patients and healthy controls. Abbreviations: C5a Complement component 5a, C5aR Complement component 5a receptor, CSU Chronic spontaneous urticaria, pg/mL picrogam/milliliter. The figure illustrates the plasma C5a **(A)** and serum C5aR level **(B)** of CSU patients compared to healthy controls.

Plasma C5a levels in CSU patients (median [IQR]: 2696 [1516–7115] pg/mL) were significantly higher than those in healthy controls (1764.5 [1391–2640] pg/mL), (p = 0.027) (Fig 1A). Moreover, plasma C5a levels in severe CSU patients (7115 [3498–32934] pg/mL) were significantly increased compared to non-severe CSU patients (1705 [1338–2499] pg/mL), (p < 0.001) and healthy controls (1764.5 [1391–2640] pg/mL, p < 0.001). Nonetheless, there was no statistically significant difference in plasma C5a levels between non-severe CSU patients and healthy controls (p = 0.75) (S1 Fig).

Serum C5aR levels in CSU patients (351 [142–704] pg/mL) were significantly lower than those in healthy controls (1682 [433–2732] pg/mL), (p < 0.001) (Fig 1B). Although the serum C5aR levels in the severe CSU patients (324 [148–976] pg/mL) and non-severe CSU groups (357 [128–625] pg/mL) were both significantly decreased compared to those in the control group (1682 [433–2732] pg/mL) (all p < 0.001), we did not observe a significant difference in serum C5aR levels between the severe and non-severe CSU groups (p = 0.748) (S2 Fig).

**Fig 2:**
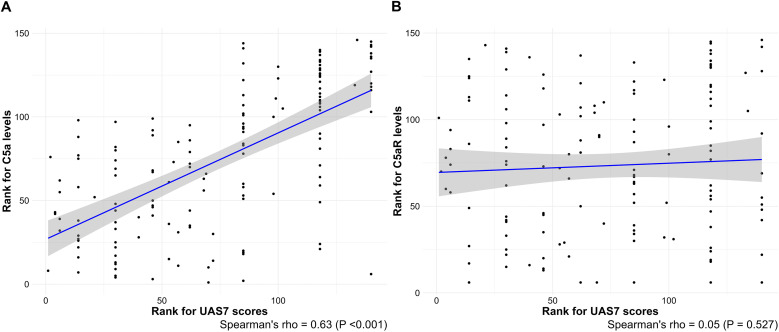
Correlation between levels of C5a (A) and C5aR (B) and UAS7 scores among CSU patients. Abbreviations: C5a complement component 5a, C5aR complement component 5a receptor, UAS7 Urticaria activity score over 7 days. Spearman’s rank correlation analyses show the correlation between the disease activity of CSU and C5a level **(A)** or C5aR level **(B).**

To examine the association between the disease activity of CSU and the levels of C5a and C5aR, we conducted Spearman’s correlation analyses, which revealed a strong association between the plasma C5a titer and CSU activity based on the UAS7 score (rho = 0.63, p < 0.001), while no relation to the C5aR level was found (rho = 0.05, p = 0.527) (Fig 2).

Univariate and multivariate logistic regression analysis, which was adjusted for demographic and disease-related variables, further supported the association between elevated C5a levels and severe urticaria (CSU), compared to the non-severe group, with the detailed findings of logistic regression shown in Table 2.

**Table 2:** Logistic regression analysis of factors associated with severe chronic spontaneous urticaria.

Variables	Univariable analysis		Multivariable analysis^a^	
	OR (95% CI)	*p*	aOR (95% CI)	*p*
**C5a** (10-unit increase)	**1.01 (1.004-1.011)**	**<0.001**	**1.01 (1.01-1.02)**	**<0.001**
**C5aR** (10-unit increase)	1.00 (0.998-1.01)	0.250	0.99 (0.98-1.01)	0.307
**C5a** (log-transformed, 1-SD increased)^b^	**11.84 (4.91-28.59)**	**<0.001**	**55.93 (11.05-283.13)**	**<0.001**
**C5aR** (log-transformed, 1-SD increased)^b^	1.06 (0.77, 1.47)	0.725	0.59 (0.31, 1.11)	0.100

Abbreviations: OR odds ratio, aOR adjusted odds ratio.

^a^Adjusted for sex, age, disease duration, angioedema, elevated C-reactive protein level, increased IgE, eosinopenia, increased IgG anti-TPO, and D-dimer. Sufficient findings are shown in **S1 Table**.

^b^The variable was log-transformed and included in separate multivariable models from C5a and C5aR.

**Fig 3:**
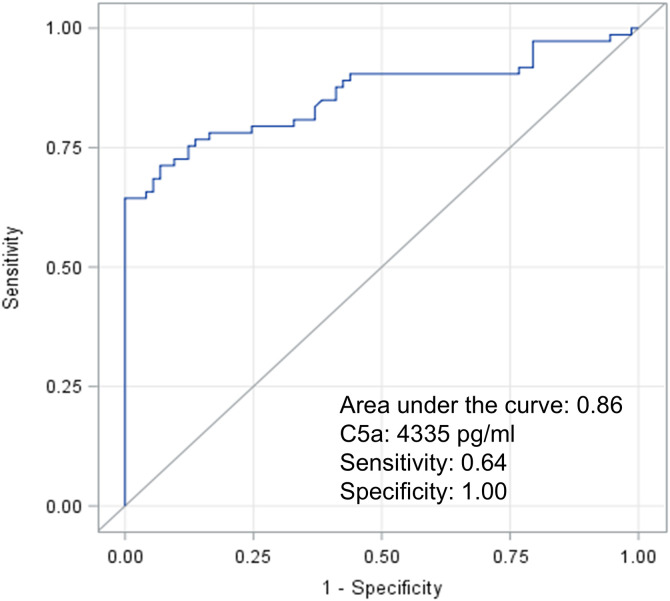
ROC curve for C5a in differentiating disease severity. Abbreviations: C5a complement component 5a, pg/mL picrogam/milliliter.

The ROC analysis identified a C5a level of 4335 pg/mL serving as the optimal threshold for indicating disease severity in the study cohort, with the area under the curve (AUC) of 0.86 (95% CI: 0.80–0.92, p < 0.001), the sensitivity of 64% (95% CI: 52% – 75%), and the specificity of 100% (95% CI: 95% – 100%) (Fig 3).

### Factors related to plasma C5a and serum C5aR levels in CSU patients

We investigated possible factors associated with the level of C5a and C5aR in CSU patients, employing appropriately the Mann-Whitney U test and Spearman’s correlation analyses. While plasma C5a levels were significantly higher in female patients, and those with a family history of CSU, no differences were found for other characteristics, including angioedema, disease duration, ANA, and ASST status. We did not observe the significant difference between C5aR levels and other baseline factors based on comparative Mann-Whitney U test (Table 3). Spearman’s correlation analyses found a weak correlation between plasma C5a levels with age of patient, white blood cell count (WBC), ERS, PT, and IgG anti-TPO. In contrast, serum C5aR levels in CSU patients demonstrated a weak correlation with eosinophil count, ERS, and IgG anti-TPO levels (Table 4). After multiple testing correction, age and WBC remained associated with C5a, whereas no examined factors showed a clear correlation with C5aR (S2 Table).

**Table 3:** Comparative analyses examining the association between C5a and C5aR levels with baseline characteristics of CSU patients.

Variables	C5a (pg/mL)	p	C5aR (pg/mL)	p
	Median [IQR]		Median [IQR]	
**Gender**				
Male (n = 54)	1771 [1222 - 4147]	**0.003**	333.5 [153 - 490]	0.392
Female (n = 92)	3531.5 [1817.5 - 11107.5]		365.5 [142–973]	
**Family history of urticaria**				
No (n = 132)	2503.5 [1475–5741]	**0.020**	336 [142–696]	0.098
Yes (n = 14)	4246 [2106–39683]		587 [250–1208]	
**Angioedema**				
No (n = 92)	2794 [1608 - 5191.5]	0.906	368 [172–696]	0.303
Yes (n = 54)	2451.5 [1408–9316]		282 [48–970]	
**Disease duration**				
<1 year (n = 48)	3451.5 [1430 - 8256.5]	0.998	317 [129.5 - 623.5]	0.463
≥1 year (n = 98)	2503.5 [1594 - 6001]		354.5 [153–704]	
**ANA**				
Negative (n = 128)	2556 [1583.5, 7156]	0.976	351 [142, 696]	0.375
Positive (n = 18)	3331 [1318, 5031]		352.5 [250, 1282]	
**ASST**				
Negative (n = 49)	3022 [1665, 4470]	0.862	368 [172, 970]	0.443
Positive (n = 97)	2499 [1485, 7197]		316 [142, 694]	

Abbreviations/: C5a complement component 5a, C5aR complement component 5a receptor, pg/mL picrogam/milliliter, IQR interquartile range, ANA antinuclear antibodies, ASST autologous serum skin test.

All analyses were performed using Mann-Whitney U test.

**Table 4:** Correlation analyses examining the correlation between plasma C5a and serum C5aR concentration with baseline characteristics of CSU patients.

Variables	C5a		C5aR	
	r^a^	*p*	r^a^	*p*
C5aR	0.14	0.085	–	*–*
Age	0.23	**0.002**	0.12	0.114
D-Dimer	0.04	0.662	0.02	0.776
WBC	0.25	**0.003**	0.04	0.639
Eosinophils	0.06	0.479	0.17	**0.039**
Basophils	0.02	0.801	−0.01	0.884
IgE	0.01	0.931	0.13	0.113
CRP	0.10	0.211	0.10	0.236
ESR	0.21	**0.010**	0.19	**0.019**
IgG anti-TPO	0.19	**0.024**	0.19	**0.020**
PT (s)	−0.19	**0.024**	−0.06	0.457
PT (%)	0.19	**0.023**	0.06	0.456
APTT (s)	−0.03	0.755	0.06	0.490
APTT (%)	−0.002	0.978	0.07	0.399
Fibrinogen	0.12	0.143	0.06	0.469

Abbreviations: C5a complement component 5a, C5aR complement component 5a receptor, WBC IgE immunoglobulin E, CRP C-reactive protein, ESR erythrocyte sedimentation rate, CRP C-reactive protein, IgG immunoglobulin G, TPO Thyroid Peroxidase, PT prothrombin time, APTT activated partial thromboplastin time.

^a^Spearman’s correlation analysis.

## Discussion

Our findings revealed that plasma C5a levels in patients with CSU were significantly higher, while serum C5aR levels were decreased compared to matched healthy controls. The finding pertaining to C5a is consistent with prior results reported by Zhu (2012) [12], Alizadeh (2021) [17], and Bhatia (2024) [18], which suggested that C5a play a significant role in the pathogenesis of CSU. However, upon stratifying by disease severity, our study found that plasma C5a levels were elevated exclusively in the severe CSU group compared to both the non-severe CSU group and healthy donors, with no statistically significant difference observed between the non-severe CSU group and the control group. This unique finding indicates that the role of C5a may be associated with the severe subset of CSU patients. Our study furthermore elucidated the role of C5a level in the indication of CSU severity. Although the study by Bhatia et al. involving 82 CSU patients did not demonstrate a correlation between C5a levels and disease severity [18], our investigation in a larger cohort of Southeast Asian patients, an underrepresented population, highlighted a strong positive correlation between plasma C5a levels and UAS7, an internationally accepted, standardized scoring system. These findings further support the hypothesis that C5a contributes to the underlying mechanism of CSU. In addition, our findings infer that a threshold of 4335 pg/mL may be clinically applicable, which prompts earlier and more extensive management.

To our knowledge, this is the first study to quantify serum levels of soluble C5aR in CSU patients. Notably, the results demonstrated that serum C5aR concentrations were significantly lower in CSU patients compared to healthy adult controls. This reduction may be explained by the increase in plasma C5a levels, as its binding to C5aR leads to receptor neutralization or downregulation. The dynamic equilibrium between circulating C5aR and its membrane-bound form may account for the decreased serum levels. Previous studies have indicated a hypothesis that upon binding of C5a to its receptor C5aR, the receptor–agonist complex undergoes rapid internalization into endosomes within 10–15 minutes, shifting receptor presence from the extracellular to intracellular compartments [24]. Similarly, in conditions such as sepsis and septic shock, elevated C5a levels are associated with a decreased expression of C5aR on the surface of neutrophils and monocytes, underpinning the proposal mechanism of internalization [23,25]. However, no statistically significant difference in C5aR levels was observed between patients with severe and non-severe CSU, indicating that C5aR may serve more as a diagnostic marker than a prognostic one. Moreover, no statistically significant correlation between plasma C5a and serum C5aR concentrations in CSU patients shows the complexity of C5aR dynamics. Therefore, further studies are warranted to investigate the expression of C5aR at the cellular level, which may provide deeper insights into its role in CSU pathophysiology and its potential as a therapeutic target.

The observed variation of C5a and C5aR levels among CSU patients is suggested to be driven from enhanced activation of the coagulation system, which has been evidenced by several key findings from prior studies. Markers of coagulation activation such as prothrombin fragment 1 + 2 (F1 + 2) and thrombin-antithrombin complex (TAT) are significantly elevated in CSU patients compared to healthy controls [10–13]. Likewise, D-dimer, a fibrinolytic byproduct that indirectly reflects increased coagulation activity, is also elevated in CSU and has been shown to correlate with disease severity [26–29]. Moreover, some studies have reported some clinical improvement in CSU patients treated with anticoagulant agents such as heparin or warfarin [30,31]. Further supporting this link, the level of activated factor VII (FVIIa) has been found to be significantly elevated in patients with CSU [10], whereas no corresponding increase in activated factor XII (FXIIa), an essential hallmark of the intrinsic pathway, has been observed [10,14]. Contributing to this speculation, tissue factor (TF), the primary trigger of the extrinsic coagulation pathway, has been found to be overexpressed on the surface of endothelial cells and mononuclear cells in CSU patients [32]. These findings suggest that the activation of the extrinsic coagulation pathway plays a profound role in the evolving pathogenesis of CSU, besides the established mechanisms involving the FcεRI or MRGPRX2. Yanase *et al*. demonstrated that activated coagulation factors can induce the generation of C5a and C3a, which subsequently stimulate mast cells and basophils to activate and degranulate via their respective receptors, C5aR and C3aR [13]. Although both mast cells and basophils express C3aR and C5aR on their surface, mast cells are primarily activated by C5a via C5aR, with only limited activation by C3a through C3aR [16]. Similarly, only C5a can induce the degranulation of basophils through C5aR, whereas C3a exerts minimal effect [16]. Altogether, these findings suggest a hypothesis that the activation of extrinsic coagulation pathway, which generates activated coagulation factors such as FVIIa, FIIa, and FXIIIa, leads to complement activation and the release of C5a. C5a then activates mast cells and basophils via C5aR, playing a critical role in the pathogenesis of CSU. This mechanistic insight may warrant further research into C5aR antagonists as a potential therapeutic approach for chronic urticaria [16].

In addition to characterizing alterations in C5a and C5aR levels in CSU patients, our study identified several clinical and laboratory parameters associated with these biomarkers. Plasma C5a concentrations were significantly correlated with age, sex, family history of CSU, leukocyte count, erythrocyte sedimentation rate, IgG anti-TPO levels, and, notably, PT. In contrast, serum C5aR levels were associated with eosinophil count, erythrocyte sedimentation rate, and IgG anti-TPO levels. Although there is a statistical association between plasma C5a levels and CSU severity, and both parameters are significantly increased in older and female patients, establishing a causal relationship warrants further studies. Higher plasma C5a levels observed in female CSU patients can be explained by higher frequency of Type IIb endotype CSU among females [33]. Importantly, we found a significant correlation between plasma C5a concentration and prothrombin time, a laboratory parameter reflecting the activity of the extrinsic coagulation pathway, albeit with previous studies reporting enhanced activation of the extrinsic coagulation cascade in CSU, leading to secondary complement activation and generation of C5a [13,15]. These findings reinforce the hypothesis of cross-talk between the coagulation and complement systems in the pathogenesis of CSU. Furthermore, we identified a correlation between the levels of C5a/C5aR with IgG anti-TPO levels, an autoantibody linked to autoimmune urticaria [7]. These associations are compatible with the notion that immune complexes can activate the complement system through the classical pathway, thereby promoting the release of C5a. Collectively, these results suggest that in CSU, C5a may be generated through both classical pathway activation by immune complexes and coagulation–complement interactions, which further highlights the multifaceted nature of the CSU immunopathogenesis.

Despite these insights, our study has several limitations inherent to its cross-sectional design. While focusing on circulating levels, C5aR expression on blood and mast cells are not evaluated, which limits our understanding of the dynamic equilibrium between soluble and membrane-bound C5aR. The relatively small size of the control group and the limited characterization of its participants may reduce methodological rigor and constrain the robustness of the findings. In addition, the relatively modest number of CSU patients raises concerns about potential overfitting in the ROC models and the limited diversity of medical treatments, particularly the absence of biologic agents, may reduce the generalizability of our findings. Moreover, evaluating tissue-level C5a concentrations, C5aR expression on skin-resident mast cells, and their interactions with other immune components could provide deeper insights into CSU pathogenesis. These unmet gaps underscore the need for comprehensive longitudinal studies with larger and more diverse cohorts to elucidate the role of complement–coagulation interplay and receptor dynamics in CSU.

## Conclusions

Plasma levels of C5a were significantly elevated in patients with CSU compared to healthy controls and may serve as an indicative biomarker of disease severity. In contrast, serum C5aR levels were significantly lower in CSU patients compared to healthy controls but did not demonstrate a correlation with disease severity. Further studies are needed to investigate the C5a levels and C5aR expression levels in both blood and skin lesion of CSU patients to more accurately determine its role in the pathogenesis of CSU.

## Supporting information

S1 Fig Levels of C5a (A) Severe CSU patients vs. healthy controls, (B) Non-severe CSU patients vs. healthy controls, and (C) severe vs. non-severe CSU patients. (PNG)

S2 Fig Levels of C5aR (A) Severe CSU patients vs. healthy controls, (B) Non-severe CSU patients vs. healthy controls, and (C). (PNG)

S1 Table Logistic regression analysis of factors associated with severe Chronic spontaneous urticaria. (DOCX)

S2 Table Correlation between plasma C5a and serum C5aR concentrations with baseline characteristics of CSU patients corrected for multiple testing. (DOCX)
