# Supplementary figures and images for: Plasma C5a and serum C5aR levels in patients with chronic spontaneous urticaria: A single-center case-control study
Source: PLoS One. 2026 Jun 26;21(6):e0351329. doi: 10.1371/journal.pone.0351329 (PMC13308836; doi:10.1371/journal.pone.0351329)

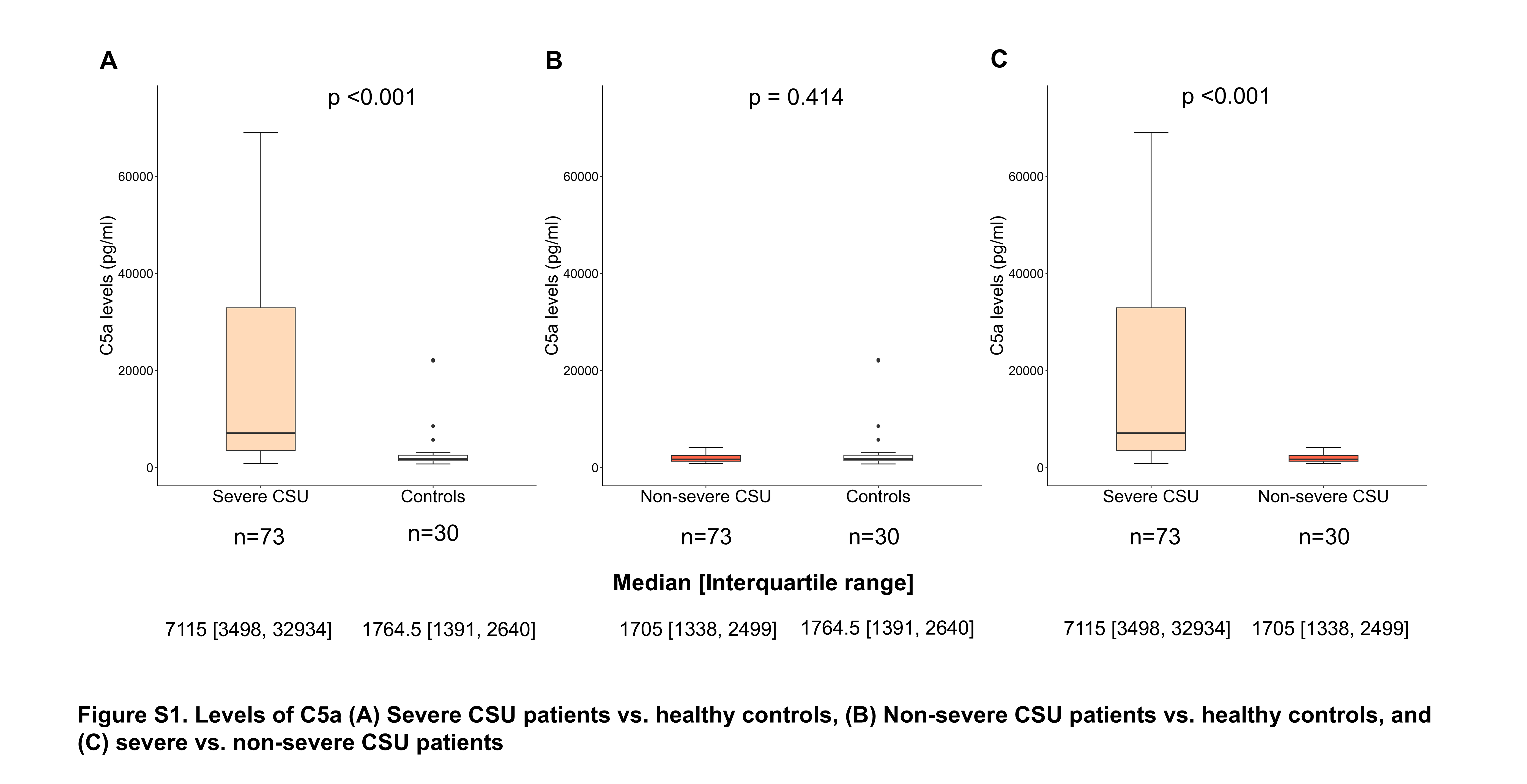

Supplement: S1 Fig — (PNG) [file pone.0351329.s001.png]

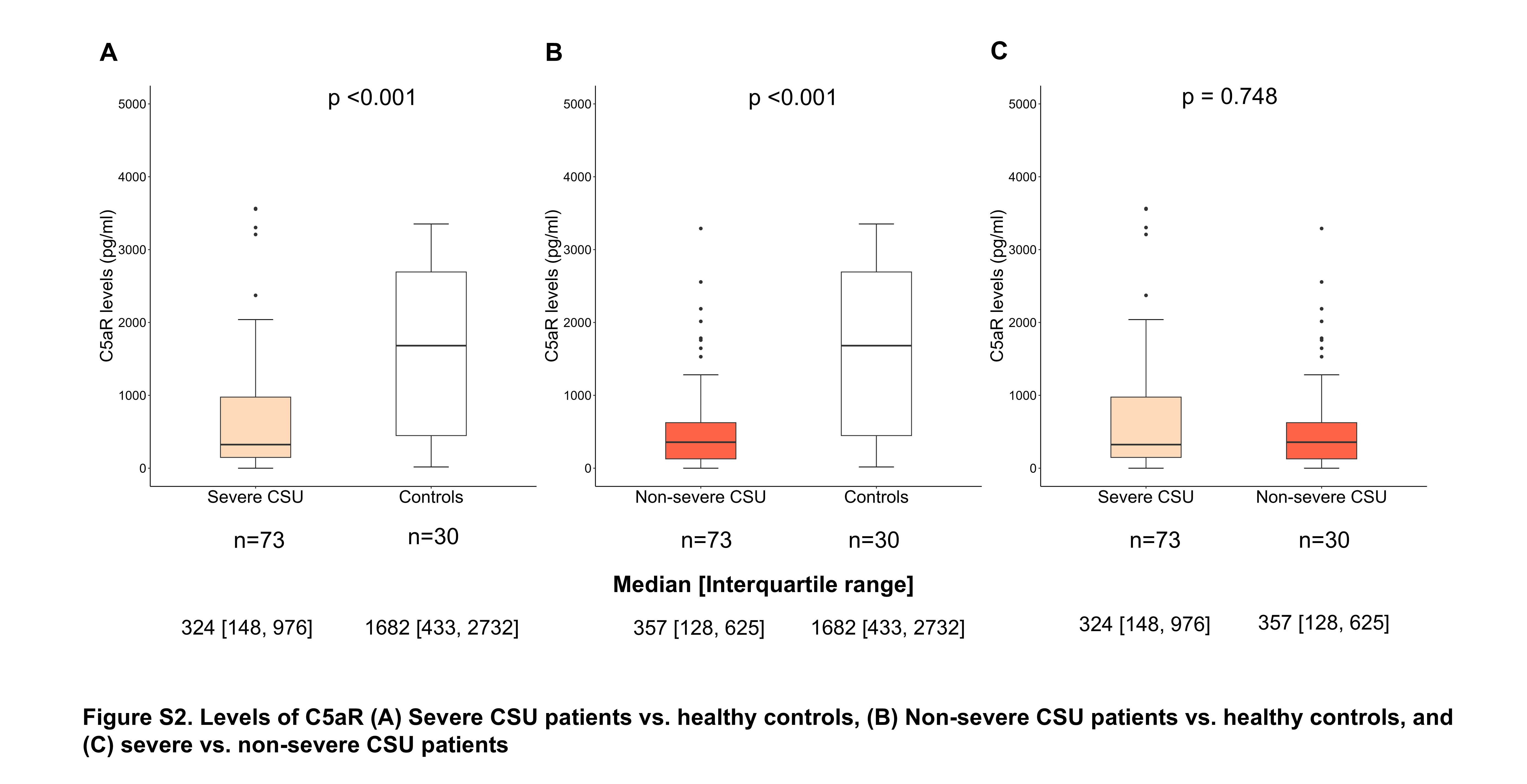

Supplement: S2 Fig — (PNG) [file pone.0351329.s002.png]
