# Supplementary material for: Plasma C5a and serum C5aR levels in patients with chronic spontaneous urticaria: A single-center case-control study
Source: PLoS One. 2026 Jun 26;21(6):e0351329. doi: 10.1371/journal.pone.0351329 (PMC13308836; doi:10.1371/journal.pone.0351329)
Supplement: S1 Table — (DOCX) [file pone.0351329.s003.docx]

**Table S1.** Logistic regression analysis of factors associated with severe chronic spontaneous urticaria

| **Variables** | **Univariable analysis** | | **Multivariable analysis** | |
| --- | --- | --- | --- | --- |
|  | **OR (95% CI)** | ***p*** | **aOR (95% CI)** | ***p*** |
| Sex (men) | 0.62 (0.32, 1.23) | 0.171 | 1.05 (0.26, 4.25) | 0.948 |
| Age at study entry | **1.05 (1.02, 1.09)** | **0.001** | 1.05 (0.99, 1.12) | 0.110 |
| Disease duration | 1.00 (0.99, 1.01) | 0.680 | 0.99 (0.96, 1.02) | 0.360 |
| Angioedema | 1.00 (0.51, 1.96) | 1.000 | 0.39 (0.10, 1.53) | 0.178 |
| Elevated CRP | **5.54 (1.52, 20.2)** | **0.010** | 2.84 (0.32, 25.14) | 0.348 |
| IgE (>100) | 0.93 (0.45, 1.92) | 0.854 | 0.35 (0.09, 1.37) | 0.132 |
| Eosinopenia | 1.26 (0.49, 3.26) | 0.631 | 0.42 (0.06, 2.82) | 0.375 |
| Elevated IgG anti- TPO | 2.61 (0.49, 13.9) | 0.261 | 0.63 (0.003, 112.45) | 0.859 |
| D-dimer (10-unit increase) | **1.007 (1.003, 1.011)** | **0.001** | **1.02 (1.01, 1.02)** | **<0.0001** |
| **C5a** (10-unit increase) | **1.008 (1.004, 1.011)** | **<0.0001** | **1.01 (1.01, 1.02)** | **<0.0001** |
| **C5aR** (10-unit increase) | 1.003 (0.998, 1.007) | 0.250 | 0.99 (0.98, 1.01) | 0.307 |
| **C5a** (1-SD increase, log-transformed)^1^ | **11.84 (4.91, 28.59)** | **<0.0001** | **55.93 (11.05, 283.13)** | **<0.0001** |
| **C5aR** (1-SD increase, log-transformed)^1^ | 1.06 (0.77, 1.47) | 0.725 | 0.59 (0.31, 1.11) | 0.100 |

Abbreviations: OR odds ratio, aOR adjusted odds ratio, CRP C-reactive protein, IgE immunoglobulin E, IgG immunoglobulin G, TPO Thyroid Peroxidase, C5a complement component 5a.

^1^ The variable was log-transformed and included in separate multivariable models from C5a and C5aR.
