# Supplementary material for: Plasma C5a and serum C5aR levels in patients with chronic spontaneous urticaria: A single-center case-control study
Source: PLoS One. 2026 Jun 26;21(6):e0351329. doi: 10.1371/journal.pone.0351329 (PMC13308836; doi:10.1371/journal.pone.0351329)
Supplement: S2 Table — (DOCX) [file pone.0351329.s004.docx]

**Table S2.** Correlation between plasma C5a and serum C5aR concentrations with baseline characteristics of CSU patients corrected for multiple testing

| **Variables** | **C5a** | | | | **C5aR** | | |
| --- | --- | --- | --- | --- | --- | --- | --- |
|  | r^a^ | *p* | *p* (adjusted)^b^ | r^a^ | | *p* | *p* (adjusted)^b^ |
| C5aR | 0.14 | 0.085 | 0.182 | - | | *-* | *-* |
| Age | 0.23 | **0.002** | **0.030** | 0.12 | | 0.114 | 0.285 |
| D-Dimer | 0.04 | 0.662 | 0.903 | 0.02 | | 0.776 | 0.831 |
| WBC | 0.25 | **0.003** | **0.023** | 0.04 | | 0.639 | 0.737 |
| Eosinophils | 0.06 | 0.479 | 0.719 | 0.17 | | **0.039** | 0.195 |
| Basophils | 0.02 | 0.801 | 0.924 | -0.01 | | 0.884 | 0.884 |
| IgE | 0.01 | 0.931 | 0.998 | 0.13 | | 0.113 | 0.339 |
| CRP | 0.10 | 0.211 | 0.352 | 0.10 | | 0.236 | 0.506 |
| ESR | 0.21 | **0.010** | 0.050 | 0.19 | | **0.019** | 0.285 |
| IgG anti-TPO | 0.19 | **0.024** | 0.072 | 0.19 | | **0.020** | 0.150 |
| PT (s) | -0.19 | **0.024** | 0.072 | -0.06 | | 0.457 | 0.686 |
| PT (%) | 0.19 | **0.023** | 0.086 | 0.06 | | 0.456 | 0.760 |
| APTT (s) | -0.03 | 0.755 | 0.944 | 0.06 | | 0.490 | 0.613 |
| APTT (%) | -0.002 | 0.978 | 0.978 | 0.07 | | 0.399 | 0.748 |
| Fibrinogen | 0.12 | 0.143 | 0.268 | 0.06 | | 0.469 | 0.640 |

Abbreviations: C5a complement component 5a, C5aR complement component 5a receptor, WBC IgE immunoglobulin E, CRP C-reactive protein, ESR erythrocyte sedimentation rate, CRP C-reactive protein, IgG immunoglobulin G, TPO Thyroid Peroxidase, PT prothrombin time, APTT activated partial thromboplastin time.

^a^ Spearman’s correlation analysis.

^b^ *p*-values corrected for multiple testing using the Benjamini–Hochberg procedure.
