# Supplementary material for: Plasma C5a and serum C5aR levels in patients with chronic spontaneous urticaria: A single-center case-control study
Source: PLoS One. 2026 Jun 26;21(6):e0351329. doi: 10.1371/journal.pone.0351329 (PMC13308836; doi:10.1371/journal.pone.0351329)
Supplement: Attachment — Submitted filename: R2. Response to Reviewer.docx [file pone.0351329.s008.docx]

**RESPONSE TO REVIEWERS**

**Article title:** Plasma C5a and serum C5aR levels in patients with chronic spontaneous urticaria: A single-center case-control study

**Short title:** C5a and C5aR in chronic spontaneous urticaria

**Authors:** Thao T. Pham, MD^1,2^, Minh N. Vu, MD, PhD^1,2^, My H. Le, MD, PhD^2^, Doanh H. Le, MD, PhD^1,2^, Thuong V. Nguyen, MD, PhD^1,2^, Phuong T.M. Pham, MD, PhD^2^

**Affiliations:**

^1^ Department of Dermatology, Hanoi Medical University, Hanoi, Vietnam

^2^ National Hospital of Dermatology and Venereology, Hanoi, Vietnam

**Corresponding author:** Phuong T.M. Pham, MD, PhD; National Hospital of Dermatology and Venereology, Hanoi, Vietnam; Email: phuongphamdv@gmail.com

**Additional Editor Comments:

I have completed a second ground's reviewing because your study can be documented (by editorial point) due to its relevant results in this specific field.
I would be happy if you could carefully re-revise the revised version.
Thanks a lot
Best regards
Cheorl-Ho Kim**

*We sincerely thank Dr. Kim – the handing editor – for kind remarks and for dedicatedly reviewing our manuscript and grateful to address all concerns raised by esteemed reviewers, as outlined point-by-point below:*

**6. Review Comments to the Author**

**Reviewer #4:** **Thanks to authors for the revision. In my opionion, these results are helpful for understanding the mechanism of urticaria especially in different populations. I have no additional comments for the manuscript.**

*We greatly appreciate the reviewer’s positive remarks and recognition of the value of our study. We are pleased that the findings made contributions to the evolving understanding of the mechanisms of urticaria across different populations.*

**Reviewer #5: Dear Authors,
I have carefully reviewed the manuscript entitled “Plasma C5a and serum C5aR levels in patients with chronic spontaneous urticaria: A single-center case-control study.” This study investigates the association between circulating C5a and soluble C5aR levels and disease severity in chronic spontaneous urticaria (CSU), focusing on a Vietnamese cohort.
The topic is timely and relevant. Increasing evidence supports the interplay between complement activation, coagulation pathways, and mast cell activation in CSU pathogenesis. The authors address an important gap, particularly regarding soluble C5aR levels, which have not previously been extensively evaluated in CSU. The relatively large patient sample (n=146), use of standardized disease severity assessment (UAS7), and multivariate modeling represent strengths of the study.
However, several methodological and interpretative issues should be addressed before the manuscript can be considered for publication.**
*We thank the reviewer for the objective and constructive comments. We acknowledge that several methodological and interpretative issues required clarification, and we have carefully revised the manuscript to address these concerns. Specifically, we have provided additional methodological details, refined the statistical analyses, and moderated the mechanistic interpretations. We believe these revisions have substantially improved the clarity, rigor, and readability of our work, and we hope the revised version meets the reviewer’s expectations.*

**Major comments
1. Study Design and Control Group Size
Although the patient sample is adequate, the control group (n=30) is relatively small compared to the CSU cohort (5:1 matching). This imbalance may affect statistical robustness and ROC curve interpretation. The authors should clarify whether power analysis was performed and how matching was implemented in the statistical analysis (beyond demographic matching).**
*Thank you very much for pointing out this important consideration. We sincerely apologize for the lack of explanation regarding the matching procedure. In the revised manuscript (lines 85-86), we have clarified that, due to the limited number of healthy controls, CSU patients were grouped in sets of five with the closest age and each group was matched to one healthy donor of similar age. We agree that the 5:1 matching ratio may influence statistical robustness. Accordingly, we have explicitly acknowledged this limitation (lines 364-366) and performed additional analyses to strengthen our findings, including bootstrapping procedures for ROC curve interpretation and multiple testing correction for correlation analyses. These revisions have substantially improved the methodological clarity and scientific rigor of our work.*

**2. Pre-Analytical Conditions and Medication Washout
The manuscript states that blood sampling was performed after specific washout periods for antihistamines and immunosuppressants. The rationale for the specified washout durations (chosen according to what) should be justified. IS five days enough for the immunosuppresant especially systemic corticosteroids?**

*We thank the reviewer for this insightful observation and are pleased to elaborate on the rationale for the specified washout periods. Based on pharmacokinetics, approximately five half-lives are required for a drug to be considered cleared. In our cohort, patients were treated with bilastine or rupatadine, with half-lives of 14.5 and 5.9 hours, respectively, and thus a 5‑day washout was sufficient. For immunosuppressants, including systemic corticosteroids, cyclosporine, and methotrexate, a washout period of one month (30 days) was implemented to ensure clearance across systemic agents. These details have been clarified in the revised manuscript (lines 89-94).*

**3. Measurement of “Serum C5aR”
The biological relevance of soluble C5aR requires further clarification:
• What form of C5aR is detected by the ELISA kit (full-length receptor, shed fragment, or splice variant)?
• Is the assay validated for serum measurement?
• The discussion on receptor internalization is speculative and should be clearly presented as hypothesis rather than established mechanism.
Given that membrane-bound C5aR expression on immune cells was not assessed, the conclusions regarding receptor dynamics should be tempered.**

*We appreciate the reviewer’s insightful comment regarding the explanation and validation of the soluble C5aR assay. To ensure clarity, we have more explicitly described the assay procedures in the revised Materials and Methods section (lines 114-122) and attached the manufacturer’s documentation with this resubmission. The ELISA kits for C5a and soluble C5aR (MyBioSource) are validated for use with multiple sample types, including serum, plasma, urine, cell culture supernatants, and tissue homogenates. Since serum was used in our study, the detected C5aR represents the circulating soluble receptor, as specified by the manufacturer. All laboratory procedures—including specimen collection and storage, reagent preparation, assay execution, and data interpretation—were conducted in strict accordance with the manufacturer’s guidelines. Regarding receptor internalization, we fully agree that this remains speculative. We have revised the Discussion (lines 295-301) to present this point as a hypothesis rather than an established mechanism, and we have tempered our conclusions accordingly.*

**4. Statistical Considerations
Several statistical concerns warrant clarification:
• The authors state that linear regression was performed, yet correlation coefficients reported are Spearman’s rho. This should be corrected for clarity.
• ROC analysis reports 100% specificity. This is unusually high and raises concerns about overfitting, particularly given the modest control size. Cross-validation or bootstrapping would strengthen this analysis.
• Multiple testing correction was not mentioned despite numerous correlation analyses.**

*We greatly appreciate the reviewer’s insightful and constructive comments related to our statistical analyses. We agree that these considerations are critical to strengthening the robustness of our findings. We are pleased to address as follows:*

*-* ***Linear regression vs. correlation coefficients:*** *We apologize for the inconsistency in terminology. Since the variables analyzed were non-normally distributed, we employed Spearman’s correlation to assess associations between C5a/C5aR levels and clinical parameters. The manuscript has been revised accordingly to ensure consistent and accurate phrasing.*

*-* ***ROC analysis specificity:*** *We acknowledge the reviewer’s concern regarding the unusually high specificity observed in our ROC analysis, particularly given the modest control size. To address this, we performed bootstrapping procedures to evaluate the robustness of the ROC estimates. The bootstrapped results confirmed high specificity, but with confidence intervals reflecting the limited control sample size. It is important to note that the specificity of 100% does not imply 100% certainty. It only reflects that zero false positives occurred in our sample. These details have been incorporated into the revised Methods and Results (lines 149-151 and 234-235), and the Discussion (lines 366-367 and 371-374) now explicitly acknowledges the potential for overfitting and the need for validation in larger, independent cohorts.*

*-* ***Multiple testing correction for correlation analyses:*** *Multiple testing correction: We appreciate the reviewer’s emphasis on controlling for type I error in the context of multiple correlation analyses. In the revised manuscript, we have specified that the Benjamini–Hochberg procedure was applied to adjust p-values across all correlation tests. Corrected p-values are now reported in Table S2, and the Methods section has been updated accordingly (lines 151-154).*

*We believe these revisions substantially improve the methodological clarity, transparency, and statistical rigor of our study.*

**5. Interpretation of Coagulation–Complement Interaction
While the mechanistic discussion linking extrinsic coagulation activation to complement activation is comprehensive, it is largely inferential in this study. Since direct markers such as F1+2, TAT, or FVIIa were not measured, the authors should avoid overextending mechanistic conclusions beyond the observed associations.**

*We appreciate the reviewer’s insightful comment regarding the interpretation of coagulation–complement interactions. We agree that our mechanistic discussion was largely inferential, as direct markers such as F1+2, TAT, or FVIIa were not measured in this study. In response, we have revised the Discussion to temper our language, clearly distinguishing observed associations from mechanistic speculation (Paragraph lines 309-337 where highlighted). We now emphasize that while our findings are consistent with the hypothesis of extrinsic coagulation activation contributing to complement activation, definitive mechanistic conclusions cannot be drawn without direct measurement of coagulation activation markers. We believe these revisions provide a more balanced and appropriately cautious interpretation of our results.*

**Minor Comments
1. The ellipsis (“…”) at the end of the sentence describing C5aR expression appears inappropriate in an academic manuscript. Please revise the sentence to remove the ellipsis or replace it with a precise expression (e.g., “among others”) if additional cell types are intended.**

*We thank the reviewer for pointing out the inappropriate use of an ellipsis in the description of C5aR expression. We have revised the sentence to remove the ellipsis and replaced it with a precise expression (“among other cell types”) to ensure clarity and adherence to academic style (line 75).*

**2. The description of medication management prior to blood sampling is unclear and requires clarification. The sentence currently states that samples were obtained “after at least five-day use of generation H1 antihistamines,” which is ambiguous and grammatically incorrect. It is not clear whether blood samples were collected after a defined washout period following discontinuation of these medications or after a minimum duration of use. Please clarify the medication protocol, explicitly specifying whether a washout period was applied, and indicate the exact duration of discontinuation for second-generation H1 antihistamines, immunosuppressants (particularly systemic corticosteroids), NSAIDs, and antibiotics. Given that these agents may significantly influence complement and inflammatory markers, this methodological detail is essential for accurate interpretation of the results.**

*We sincerely thank the reviewer for highlighting the need for clarification regarding medication management prior to blood sampling. In the revised manuscript, we have specified the exact washout periods applied. “Peripheral blood samples were obtained from eligible candidates, including CSU patients and healthy controls, after at least five-day discontinuation of second generation H1 antihistamines (sgAH1), one month for immunosuppressants (systemic corticosteroids, cyclosporine A, methotrexate, etc.), and one week for NSAIDs or antibiotics” (lines 89-94).*

**3. Peripheral blood samples were obtained from eligible candidates after at least five-day use of generation H1 antihistamines (sgAH1),..Here, please insert ‘second’ before ‘generation’.**

*We thank the reviewer for drawing attention to this typographical omission. The sentence has been corrected to read “second-generation H1 antihistamines (sgAH1)” in the revised manuscript. We regret the oversight and have ensured that the terminology is now consistent and accurate throughout the text.*

**4. The manuscript does not specify whether any patients were receiving omalizumab therapy at the time of enrollment or blood sampling. Given the potential immunomodulatory effects of omalizumab and its impact on disease activity and inflammatory pathways, this information is essential. Please clarify whether patients on omalizumab were included, excluded, or analyzed separately.**

*We sincerely thank the reviewer for raising this important point regarding omalizumab therapy. Omalizumab, although increasingly utilized in the management of CSU, has not been widely prescribed in our urticaria clinic due to its high financial cost and lack of insurance coverage. In practice, only a very small proportion of patients (approximately 1%) are able to access this biologic agent. Moreover, given the long half-life of omalizumab (approximately 26 days), recruitment of patients with recent exposure was not feasible. During the study period, no CSU patients with a history of omalizumab use met the eligibility criteria. We have added this clarification to the Discussion (lines 367-369, 371-374), noting that future studies with larger and more diverse patient populations—including those receiving biologic therapies—will be necessary to fully elucidate this important research question.*

**5. I suggest moving the (Patients diagnosed or suspected of having chronic inducible urticaria (CIndU) were excluded from the study) sentence to the exclusion criteria section to improve the clarity and structure of the Methods.**

*Thank you very much for this appropriate suggestion. The sentence you referred to has been relocated in the Study design and population to improve coherence and structure of the Methods. (lines 88-89).*

**6. There are several grammatical inconsistencies and typographical errors (e.g., “maching ratio,” “plasm C5a,” inconsistent spacing, and tense issues). Professional language editing is recommended.**

**7. Units should be standardized (pg/mL rather than pg/ml).**

**8. The cutoff value for C5a is inconsistently reported (4335 pg/mL in the abstract vs. 4535 pg/mL in the Results section). This discrepancy must be corrected.**

*We sincerely apologize for the typographical and grammatical inconsistencies identified in the manuscript. In response, we have conducted a thorough review and implemented comprehensive language editing to ensure clarity, accuracy, and consistency throughout the text.* *These revisions have been carefully applied to enhance the overall quality and precision of the manuscript.*

**9. Clarify whether C5a measurements were performed in duplicate and report intra-/inter-assay coefficients of variation.**

*We extremely appreciate this reviewer’s comment on the laboratory procedures in our study. To ensure the validity of the C5a and C5aR measurements, we confirm that all C5a and C5aR measurements were performed in duplicate, in accordance with the manufacturer’s instructions. The accuracy of the assay was evaluated by calculating intra-assay coefficients of variation, which were 4.2% (range 1.1% - 6.7%) for C5a. Inter-assay coefficients of variation were not determined, as the manufacturer’s protocol does not recommend or require this assessment for the kit used. We have clarified this point in the revised Methods (lines 118-122).*

**10. The term “predictive biomarker” should be used cautiously in cross-sectional case-control studies; “associated biomarker” may be more appropriate.**

*We thank the reviewer for this constructive feedback. We completely agree that findings derived from a cross-sectional study should be interpreted as associated or indicative rather than predictive or mechanistic. Accordingly, we have revised the manuscript to ensure that the conclusions are aligned with the actual robustness of our study. In addition, we have explicitly acknowledged the inherent limitations of cross-sectional evidence and have clearly stated that further longitudinal investigations are warranted.*

**11. The discussion could benefit from briefly addressing how these findings might integrate with current biologic therapies (e.g., anti-IgE therapy) in CSU.**

*We thank the reviewer for this comprehensive perspective. As noted, none of the patients in our study received biologic therapy such as omalizumab, we were therefore unable to directly evaluate the interaction between our findings and anti-IgE treatment. Nevertheless, we have acknowledged this limitation and have added a brief discussion noting that future studies should explore how C5a/C5aR pathways may integrate with current biologic therapies in CSU, as this could provide valuable insights into combined or complementary treatment strategies (line 367-369 and 371-373).*

**Overall Recommendation
This study contributes valuable data regarding complement activation in CSU and introduces novel information on circulating C5aR levels. However, important methodological clarifications, statistical refinement, and moderation of mechanistic interpretations are required.
I recommend major revision prior to reconsideration.**
*In conclusion, we sincerely appreciate the reviewer’s insightful and constructive comments. We have carefully addressed all methodological clarifications, refined the statistical analyses, and moderated the mechanistic interpretations as suggested. These revisions have substantially improved the clarity, scientific rigor, and overall readability of our manuscript.*
