# Supplementary material for: Plasma C5a and serum C5aR levels in patients with chronic spontaneous urticaria: A single-center case-control study
Source: PLoS One. 2026 Jun 26;21(6):e0351329. doi: 10.1371/journal.pone.0351329 (PMC13308836; doi:10.1371/journal.pone.0351329)
Supplement: Attachment — Submitted filename: R1. Response to Reviewer.docx [file pone.0351329.s007.docx]

**RESPONSE TO REVIEWERS**

**Article title:** Plasma C5a and serum C5aR levels in patients with chronic spontaneous urticaria: A single-center case-control study

**Short title:** C5a and C5aR in chronic spontaneous urticaria

**Authors:** Thao T. Pham, MD^1,2^, Minh N. Vu, MD, PhD^1,2^, My H. Le, MD, PhD^2^, Doanh H. Le, MD, PhD^1,2^, Thuong V. Nguyen, MD, PhD^1,2^, Phuong T.M. Pham, MD, PhD^2^

**Affiliations:**

^1^ Department of Dermatology, Hanoi Medical University, Hanoi, Vietnam

^2^ National Hospital of Dermatology and Venereology, Hanoi, Vietnam

**Corresponding author:** Phuong T.M. Pham, MD, PhD; National Hospital of Dermatology and Venereology, Hanoi, Vietnam; Email: phuongphamdv@gmail.com

**#Additional Editor Comments:**
**Thank you for your submission to our Plos One.
I have completed the review process and am pleased to inform that your study is valuable for further consideration.
As you can read the reviews, I would be happy if you appropriately revise the original manuscript.**

*We sincerely thank the Editor for considering our manuscript for further evaluation. We greatly appreciate the constructive comments provided by the reviewers, which we found immensely valuable. We have carefully addressed all their suggestions and revised the manuscript accordingly to enhance its scientific clarity, methodological transparency, and overall readability. We believe these revisions have strengthened the quality of our work, and we are grateful for the opportunity to improve our study through this process.*

**Reviewer #1: This manuscript addresses the potential role of complement components C5a and C5aR in the pathophysiology and severity of CSU. The topic is clinically relevant, as CSU remains a heterogeneous and incompletely understood condition. The work provides valuable data from a Southeast Asian population, an underrepresented group in CSU research. The manuscript is clearly written and methodologically sound. The main finding (an increase in plasma C5a levels correlating with CSU severity and a decrease in soluble C5aR) adds incremental information to the literature, particularly since a drug targeting this pathway is currently under development. Compared with previous studies by Zhu et al. (2012), Alizadeh Aghdam et al. (2021), and Bhatia et al. (2024), this manuscript provides a larger cohort, integrating both complement and coagulation pathways in a comprehensive quantitative analysis. Importantly, it is the first to measure soluble C5aR levels and to identify a plasma C5a cutoff predictive of disease severity. However, several major and minor revisions are required before the manuscript can be accepted.**

*We are grateful for your kind remarks. We greatly appreciate your recognition of our work’s contribution to the literature, particularly in highlighting data from a Southeast Asian population—an underrepresented group in pre-existing CSU research. In the revised manuscript, we have more clearly emphasized this outstanding aspect (lines 269-274) to underline the novelty and importance of our work. We have also carefully addressed the major and minor concerns raised, as detailed in our point‑by‑point responses below. These revisions were made with the aim of improving scientific clarity, methodological transparency, and overall readability.*

**Major Comments
The elevation of plasma C5a in CSU has been demonstrated in several previous studies, and one has suggested a possible link with disease severity. Please emphasize more clearly the novelty of this study, including the larger cohort size, the use of a standardized scoring system, the definition of a cutoff value, and the measurement of soluble C5aR when citing these references, as this aspect is not sufficiently highlighted in the current version**

*We sincerely thank the reviewer for drawing attention to the novel contributions of our study. We fully agree that these aspects should be emphasized more clearly. In the revised manuscript, we have explicitly highlighted the unique strengths of our work (lines 269-274). Specifically, our study includes a comparatively large cohort from a Southeast Asian population, which remains underrepresented in CSU research, together with a matched control group for comparison. We have also employed a standardized scoring system (UAS7) to assess disease severity, incorporated ROC analysis to establish a clinically relevant cutoff value for plasma C5a, and, importantly, measured soluble C5aR levels simultaneously for the first time. By underscoring these points, we aim to strengthen the methodological rigor and reaffirm the scientific novelty and robustness of our study.*

**Line 312–315: Please revise this sentence, as there is an association between C5a levels and CSU severity but no evidence of causality. Therefore, it cannot be concluded that the higher frequency of CSU in women and older patients is caused by increased C5a levels in these groups.**

*We sincerely apologize for any confusion caused by the original wording and thank the reviewer for highlighting this important point. We fully agree that, while our data demonstrate a statistical association between plasma C5a levels and CSU severity (as assessed by UAS7), this does not establish a causal relationship. Our initial intention was to describe the observation that both C5a levels and CSU severity were higher in female and older patients. However, we recognize that drawing an exposure‑to‑outcome conclusion requires much greater rigor and further investigation. Accordingly, we have revised the sentence to read: “Although there is a statistical association between plasma C5a levels and CSU severity, and both parameters are significantly increased in older and female patients, establishing a causal relationship warrants further studies.” (lines 327-331) We believe this rephrasing improves the clarity and objectivity of our interpretation.*

**Methodology: The authors did not specify whether blood samples were collected during an active phase of CSU or during remission. In addition, the sentence regarding treatments (Line 86) is unclear: was there a washout period, or were patients still receiving therapy at the time of sampling? Please clarify this point.**

*We thank the reviewer for highlighting this important point and sincerely apologize for the confusion regarding the description of treatment washout. The original wording was imprecise; our intention was to indicate that participants discontinued relevant medications prior to blood collection. The previous expression “after at least five‑day use” may have caused uncertainty for readers. We have therefore clarified the sentence as: “Peripheral blood samples were obtained from eligible candidates, including CSU patients and healthy controls, after at least five-day discontinuation of second generation H1 antihistamines (sgAH1), one month for immunosuppressant (systemic corticosteroids, cyclosporine A, methotrexate, etc.), and one week for NSAIDs or antibiotics.” (lines 87-90)*

**Minor Comments
Line 25: “matching ratio”
Line 170: Spell out “Seventy-three” at the start of the sentence.
Lines 171, 174: Harmonize p-value spacing
Line 178: Add missing space after period: “…(p < 0.001). (Fig 1B)”.
Line 176: Replace pg/ml” by pg/mL throughout.
Lines 243–244: “significantly higher.”
Line 263: Replace that by “by”**

*We sincerely thank the reviewer for carefully identifying these accuracy issues. We apologize for the errors in wording and formatting. In addition to correcting the specific points noted, we have thoroughly reviewed the entire manuscript to ensure consistency and to correct any remaining inaccuracies.*

**Reviewer #2: Review for the manuscript ,,Plasma C5a and serum C5aR levels in patients with chronic spontaneous urticaria: A single-center case-control study,,

In this review the authors tried to analyze plasma C5a and serum C5aR in Vietnamese patients with CSU and also to investigate the association with disease severity.
The results of this study suggest that plasma levels of C5a were significantly elevated in patients with CSU compared to healthy controls and may serve as a predictive biomarker of disease severity.The authors also found that plasma C5a concentrations were significantly correlated with age, sex, family, history of CSU, leukocyte count, erythrocyte sedimentation rate, IgG anti-TPO levels, and PT.
Very interesting subject with great impact od human health.**

*Thank you very much for your kind remarks and for acknowledging the significance of our work.*

**Comments
Please explain the classification of patients with a UAS7 score of 28 or higher as severe CSU, and those with a UAS7 score below 28 as non-severe CSU.
The authors should indicate the methods and equipment used to determine biochemical parameters.**

*We thank the reviewer for raising this important concern. To clarify, our urticaria clinic and this study employ the internationally accepted Urticaria Activity Score over 7 days (UAS7), which is the gold‑standard tool recommended by the EAACI/GA²LEN/EDF/WAO guidelines for assessing CSU activity. According to these guidelines, CSU severity is classified into four categories: well‑controlled (0–6), mild (7–15), moderate (16–27), and severe (≥28). In our cohort, which included patients who had discontinued relevant medications prior to sampling, approximately 50% of patients had severe CSU and 50% had mild to moderate disease. This distribution indicates a right‑skewed severity profile, with the majority of patients presenting with severe disease. As our study aimed to conduct ROC analysis to identify a predictive cut‑off value, the outcome variable needed to be binary. Therefore, we categorized patients into severe (UAS7 ≥28) and non‑severe (UAS7 <28) groups. This approach is consistent with clinical practice and enhances the robustness of statistical analysis. Furthermore, similar methodology has been adopted in previous studies, such as Lao K. et al. (Clin Exp Allergy, 2024,* [*https://onlinelibrary.wiley.com/doi/10.1111/cea.14531*](https://onlinelibrary.wiley.com/doi/10.1111/cea.14531)*) and Nguyen et al. (Sci Rep, 2025,* [*https://www.nature.com/articles/s41598-025-94841-1*](https://www.nature.com/articles/s41598-025-94841-1)*). Nevertheless, we acknowledge that further studies with larger proprietary cohorts are warranted to fully substantiate the four‑tier classification of CSU severity.*

*In addition, we have clarified the methods and equipment used to determine biochemical parameters. Levels of plasma C5a and serum C5aR, the primarily investigated parameters, were measured using commercially available ELISA kits (My BioSource, Inc, San Diego, CA, USA) according to the manufacturer’s instructions. Additional biochemical parameters, which further characterized the cohort, were quantified at the Department of Laboratory Medicine with ISO qualified. These details have been added to the Methods section (lines 104-109) to ensure transparency and reproducibility.*

**Reviewer #3: This manuscript addresses an important and timely question concerning the role of complement activation—specifically C5a and soluble C5aR—in chronic spontaneous urticaria (CSU). The topic is relevant, the patient sample is adequate for a single-center study, and the findings add data from an underrepresented population. The study demonstrates a consistent association between elevated plasma C5a levels and CSU severity, contributing to the growing literature on coagulation–complement interactions in urticaria.**

*We greatly appreciate your kind remarks and highlighting important contributions of our study. We are pleased to address the concerns you raised to enhance the scientific clarity of the manuscript.*

**However, several substantial issues limit the current quality of the manuscript and preclude acceptance in its present form. These include:
1. “There is a methodological inconsistency regarding the C5a cutoff value. The Abstract and Discussion report 4335 pg/mL, whereas the Results section and Figure 3 indicate 4535 pg/mL. The authors must clarify the correct ROC-derived cutoff and revise the manuscript accordingly.”**

*We sincerely apologize for this typographical error. We have corrected the cut-off value of C5a differentiating severe and non-severe CSU. The accurate value of* ***4335 pg/mL*** *has been revised to be consistent across the manuscript.*

**2. insufficient characterization of the control group**

*We thank the reviewer for raising this valuable observation. We fully agree that comprehensive characterization of the control group is essential to ensure the validity of comparisons between CSU patients and healthy controls. We have acknowledged this limitation in the revised manuscript (lines 348-349) to more clearly frame the interpretation of our findings. Nevertheless, the healthy controls in our study were sex- and age-matched to CSU patients and were carefully screened according to the inclusion criteria described in the Materials and Methods section (lines 87-94), which excluded individuals with prior diseases potentially affecting C5a/C5aR levels or recent use of relevant medications. While the two groups were not fully matched in terms of clinical and laboratory parameters, our approach mitigated several important confounding factors, including age, sex, concomitant medical conditions, and therapies.*

**3. limited explanation and validation of the soluble C5aR assay**

*We appreciate the reviewer’s insightful comment regarding the explanation and validation of the soluble C5aR assay. To ensure clarity, we have more explicitly described the assay procedures in the revised Materials and Methods section (lines 113-116) and have attached the original manufacturer’s documentation with this resubmission. The C5a and soluble C5aR assays employed in our study were developed by MyBioSource, a well-established provider of ELISA kits for research purposes. As detailed in the manufacturer’s protocol, soluble C5aR measurement is feasible across various sample types, including blood, urine, cell culture supernatants, and tissue homogenates. In our study, the primary objective was to assess circulating C5aR levels in patients with CSU, and all laboratory procedures—including specimen collection and storage, reagent preparation, assay execution, and data interpretation—were conducted in strict accordance with the manufacturer’s guidelines.*

**4. overinterpretation of cross-sectional data as predictive or mechanistic**

*We thank the reviewer for this constructive feedback. We completely agree that findings derived from a cross-sectional study should be interpreted as indicative rather than predictive or mechanistic. Accordingly, we have revised the manuscript (lines 44, 219, 267, 357) to ensure that the conclusions are aligned with the actual robustness of our study. In addition, we have explicitly acknowledged the inherent limitations of cross-sectional evidence and have clearly stated that further longitudinal investigations are warranted (lines 345-346, 352-354).*

**5. The manuscript reports significantly higher plasma C5a levels in female CSU patients compared to males; however, this sex difference is not addressed in the Discussion.**

*We thank the reviewer for highlighting this important point. We apologize that the sex-related difference in plasma C5a levels was not clearly addressed in the Discussion. In the revised manuscript (lines 327-331), we have explicitly discussed the significantly higher plasma C5a levels observed in female CSU patients compared to males which can be explained by higher frequency of Type IIb endotype CSU among females.*

**6. statistical concerns related to skewness, scaling of effect sizes, and multivariable modeling: No results are shown regarding regression analysis.**

*We thank the reviewer for this thoughtful consideration regarding the statistical analysis. In response, we have conducted additional analyses to rigorously evaluate the robustness of the associations and the applicability of our results. To address the skewness of C5a/C5aR levels and to minimize the influence of effect size scaling, we applied log transformation and standardized the variables by 1 SD. These adjustments allowed for clearer interpretation of the findings within the multivariable model. The revised results are now presented in Table 2, alongside the original scaling of C5a/C5aR per 10-unit increment. We believe these revisions substantially enhance the interpretability and methodological rigor of the regression analysis.*
