# Supplementary material for: Plasma C5a and serum C5aR levels in patients with chronic spontaneous urticaria: A single-center case-control study
Source: PLoS One. 2026 Jun 26;21(6):e0351329. doi: 10.1371/journal.pone.0351329 (PMC13308836; doi:10.1371/journal.pone.0351329)
Supplement: Attachment — Submitted filename: comments for authors.docx [file pone.0351329.s006.docx]

Dear Authors,

I have carefully reviewed the manuscript entitled “Plasma C5a and serum C5aR levels in patients with chronic spontaneous urticaria: A single-center case-control study.” This study investigates the association between circulating C5a and soluble C5aR levels and disease severity in chronic spontaneous urticaria (CSU), focusing on a Vietnamese cohort.

The topic is timely and relevant. Increasing evidence supports the interplay between complement activation, coagulation pathways, and mast cell activation in CSU pathogenesis. The authors address an important gap, particularly regarding soluble C5aR levels, which have not previously been extensively evaluated in CSU. The relatively large patient sample (n=146), use of standardized disease severity assessment (UAS7), and multivariate modeling represent strengths of the study.

However, several methodological and interpretative issues should be addressed before the manuscript can be considered for publication.

**Major comments**

1. Study Design and Control Group Size

Although the patient sample is adequate, the control group (n=30) is relatively small compared to the CSU cohort (5:1 matching). This imbalance may affect statistical robustness and ROC curve interpretation. The authors should clarify whether power analysis was performed and how matching was implemented in the statistical analysis (beyond demographic matching).

2. Pre-Analytical Conditions and Medication Washout

The manuscript states that blood sampling was performed after specific washout periods for antihistamines and immunosuppressants. The rationale for the specified washout durations (chosen according to what) should be justified. IS five days enough for the immunosupresant especially systemic corticosteroids?

3. Measurement of “Serum C5aR”

The biological relevance of soluble C5aR requires further clarification:

- What form of C5aR is detected by the ELISA kit (full-length receptor, shed fragment, or splice variant)?
- Is the assay validated for serum measurement?
- The discussion on receptor internalization is speculative and should be clearly presented as hypothesis rather than established mechanism.

Given that membrane-bound C5aR expression on immune cells was not assessed, the conclusions regarding receptor dynamics should be tempered.

4. Statistical Considerations

Several statistical concerns warrant clarification:

- The authors state that linear regression was performed, yet correlation coefficients reported are Spearman’s rho. This should be corrected for clarity.
- ROC analysis reports 100% specificity. This is unusually high and raises concerns about overfitting, particularly given the modest control size. Cross-validation or bootstrapping would strengthen this analysis.
- Multiple testing correction was not mentioned despite numerous correlation analyses.

5. Interpretation of Coagulation–Complement Interaction

While the mechanistic discussion linking extrinsic coagulation activation to complement activation is comprehensive, it is largely inferential in this study. Since direct markers such as F1+2, TAT, or FVIIa were not measured, the authors should avoid overextending mechanistic conclusions beyond the observed associations.

**Minor Comments**

1. The ellipsis (“…”) at the end of the sentence describing C5aR expression appears inappropriate in an academic manuscript. Please revise the sentence to remove the ellipsis or replace it with a precise expression (e.g., “among others”) if additional cell types are intended.
2. The description of medication management prior to blood sampling is unclear and requires clarification. The sentence currently states that samples were obtained “after at least five-day use of generation H1 antihistamines,” which is ambiguous and grammatically incorrect. It is not clear whether blood samples were collected after a defined washout period following discontinuation of these medications or after a minimum duration of use. Please clarify the medication protocol, explicitly specifying whether a washout period was applied, and indicate the exact duration of discontinuation for second-generation H1 antihistamines, immunosuppressants (particularly systemic corticosteroids), NSAIDs, and antibiotics. Given that these agents may significantly influence complement and inflammatory markers, this methodological detail is essential for accurate interpretation of the results.
3. Peripheral blood samples were obtained from eligible candidates after at least five-day use of generation H1 antihistamines (sgAH1),..Here, please insert ‘second’ before ‘generation’.
4. The manuscript does not specify whether any patients were receiving omalizumab therapy at the time of enrollment or blood sampling. Given the potential immunomodulatory effects of omalizumab and its impact on disease activity and inflammatory pathways, this information is essential. Please clarify whether patients on omalizumab were included, excluded, or analyzed separately.
5. I suggest moving the (Patients diagnosed or suspected of having chronic inducible urticaria (CIndU) were excluded from the study) sentence to the exclusion criteria section to improve the clarity and structure of the Methods.
6. There are several grammatical inconsistencies and typographical errors (e.g., “maching ratio,” “plasm C5a,” inconsistent spacing, and tense issues). Professional language editing is recommended.
7. Units should be standardized (pg/mL rather than pg/ml).
8. The cutoff value for C5a is inconsistently reported (4335 pg/mL in the abstract vs. 4535 pg/mL in the Results section). This discrepancy must be corrected.
9. Clarify whether C5a measurements were performed in duplicate and report intra-/inter-assay coefficients of variation.
10. The term “predictive biomarker” should be used cautiously in cross-sectional case-control studies; “associated biomarker” may be more appropriate.
11. The discussion could benefit from briefly addressing how these findings might integrate with current biologic therapies (e.g., anti-IgE therapy) in CSU.

**Overall Recommendation**

This study contributes valuable data regarding complement activation in CSU and introduces novel information on circulating C5aR levels. However, important methodological clarifications, statistical refinement, and moderation of mechanistic interpretations are required.

I recommend major revision prior to reconsideration.

Regards,

Formun Üstü

Formun Altı
