# Supplementary material for: Plasma C5a and serum C5aR levels in patients with chronic spontaneous urticaria: A single-center case-control study
Source: PLoS One. 2026 Jun 26;21(6):e0351329. doi: 10.1371/journal.pone.0351329 (PMC13308836; doi:10.1371/journal.pone.0351329)
Supplement: Peer review history

# Peer review: Decision Letter 0

**DOI:** 10.1371/journal.pone.0351329.r001

**Authors:** Cheorl-Ho Kim

13 Dec 2025

PONE-D-25-49336

Plasma C5a and serum C5aR levels in patients with chronic spontaneous urticaria: A single-center case-control study

PLOS One

Dear Dr. Nguyen,

Thank you for submitting your manuscript to PLOS ONE. After careful consideration, we feel that it has merit but does not fully meet PLOS ONE’s publication criteria as it currently stands. Therefore, we invite you to submit a revised version of the manuscript that addresses the points raised during the review process.

Please submit your revised manuscript by Jan 27 2026 11:59PM. If you will need more time than this to complete your revisions, please reply to this message or contact the journal office at plosone@plos.org. When you're ready to submit your revision, log on to https://www.editorialmanager.com/pone/ and select the 'Submissions Needing Revision' folder to locate your manuscript file.

Please include the following items when submitting your revised manuscript:

- A letter that responds to each point raised by the academic editor and reviewer(s). You should upload this letter as a separate file labeled 'Response to Reviewers'.
- A marked-up copy of your manuscript that highlights changes made to the original version. You should upload this as a separate file labeled 'Revised Manuscript with Track Changes'.
- An unmarked version of your revised paper without tracked changes. You should upload this as a separate file labeled 'Manuscript'.

If you would like to make changes to your financial disclosure, please include your updated statement in your cover letter. Guidelines for resubmitting your figure files are available below the reviewer comments at the end of this letter.

If applicable, we recommend that you deposit your laboratory protocols in protocols.io to enhance the reproducibility of your results. Protocols.io assigns your protocol its own identifier (DOI) so that it can be cited independently in the future. For instructions see: https://journals.plos.org/plosone/s/submission-guidelines#loc-laboratory-protocols. Additionally, PLOS ONE offers an option for publishing peer-reviewed Lab Protocol articles, which describe protocols hosted on protocols.io. Read more information on sharing protocols at https://plos.org/protocols?utm_medium=editorial-email&utm_source=authorletters&utm_campaign=protocols.

We look forward to receiving your revised manuscript.

Kind regards,

Cheorl-Ho Kim, Ph.D.

Academic Editor

PLOS One

**Journal Requirements:**

When submitting your revision, we need you to address these additional requirements.

1. Please ensure that your manuscript meets PLOS ONE's style requirements, including those for file naming. The PLOS ONE style templates can be found at

https://journals.plos.org/plosone/s/file?id=wjVg/PLOSOne_formatting_sample_main_body.pdf and

https://journals.plos.org/plosone/s/file?id=ba62/PLOSOne_formatting_sample_title_authors_affiliations.pdf

2. In the online submission form you indicate that your data is not available for proprietary reasons and have provided a contact point for accessing this data. Please note that your current contact point is a co-author on this manuscript. According to our Data Policy, the contact point must not be an author on the manuscript and must be an institutional contact, ideally not an individual. Please revise your data statement to a non-author institutional point of contact, such as a data access or ethics committee, and send this to us via return email. Please also include contact information for the third party organization, and please include the full citation of where the data can be found.

3. Please upload a new copy of Figures 1, and 3; and Supplementary Figures 1 and 2 as the detail is not clear. Please follow the link for more information:  https://journals.plos.org/plosone/s/figures

4. If the reviewer comments include a recommendation to cite specific previously published works, please review and evaluate these publications to determine whether they are relevant and should be cited. There is no requirement to cite these works unless the editor has indicated otherwise.

**Additional Editor Comments:**

Thank you for your submission to our Plos One.

I have completed the review process and am pleased to inform that your study is valuable for further consideration.

As you can read the reviews, I would be happy if you appropriately revise the original manuscript.

Thank you

Sincerely,

Cheorl-Ho Kim Ph.D

Editor

[Note: HTML markup is below. Please do not edit.]

Reviewers' comments:

Reviewer's Responses to Questions

**Comments to the Author**

1. Is the manuscript technically sound, and do the data support the conclusions?

The manuscript must describe a technically sound piece of scientific research with data that supports the conclusions. Experiments must have been conducted rigorously, with appropriate controls, replication, and sample sizes. The conclusions must be drawn appropriately based on the data presented.

Reviewer #1: Yes

Reviewer #2: Yes

Reviewer #3: Yes

**********

2. Has the statistical analysis been performed appropriately and rigorously?

Reviewer #1: Yes

Reviewer #2: Yes

Reviewer #3: No

**********

3. Have the authors made all data underlying the findings in their manuscript fully available?

The PLOS Data policy requires authors to make all data underlying the findings described in their manuscript fully available without restriction, with rare exception (please refer to the Data Availability Statement in the manuscript PDF file). The data should be provided as part of the manuscript or its supporting information, or deposited to a public repository. For example, in addition to summary statistics, the data points behind means, medians and variance measures should be available. If there are restrictions on publicly sharing data—e.g. participant privacy or use of data from a third party—those must be specified.

Reviewer #1: Yes

Reviewer #2: Yes

Reviewer #3: No

**********

4. Is the manuscript presented in an intelligible fashion and written in standard English?

PLOS ONE does not copyedit accepted manuscripts, so the language in submitted articles must be clear, correct, and unambiguous. Any typographical or grammatical errors should be corrected at revision, so please note any specific errors here.

Reviewer #1: Yes

Reviewer #2: Yes

Reviewer #3: Yes

**********

5. Review Comments to the Author

Please use the space provided to explain your answers to the questions above. You may also include additional comments for the author, including concerns about dual publication, research ethics, or publication ethics. (Please upload your review as an attachment if it exceeds 20,000 characters)

Reviewer #1: This manuscript addresses the potential role of complement components C5a and C5aR in the pathophysiology and severity of CSU. The topic is clinically relevant, as CSU remains a heterogeneous and incompletely understood condition. The work provides valuable data from a Southeast Asian population, an underrepresented group in CSU research. The manuscript is clearly written and methodologically sound. The main finding (an increase in plasma C5a levels correlating with CSU severity and a decrease in soluble C5aR) adds incremental information to the literature, particularly since a drug targeting this pathway is currently under development. Compared with previous studies by Zhu et al. (2012), Alizadeh Aghdam et al. (2021), and Bhatia et al. (2024), this manuscript provides a larger cohort, integrating both complement and coagulation pathways in a comprehensive quantitative analysis. Importantly, it is the first to measure soluble C5aR levels and to identify a plasma C5a cutoff predictive of disease severity. However, several major and minor revisions are required before the manuscript can be accepted.

Major Comments

The elevation of plasma C5a in CSU has been demonstrated in several previous studies, and one has suggested a possible link with disease severity. Please emphasize more clearly the novelty of this study, including the larger cohort size, the use of a standardized scoring system, the definition of a cutoff value, and the measurement of soluble C5aR when citing these references, as this aspect is not sufficiently highlighted in the current version

Line 312–315: Please revise this sentence, as there is an association between C5a levels and CSU severity but no evidence of causality. Therefore, it cannot be concluded that the higher frequency of CSU in women and older patients is caused by increased C5a levels in these groups.

Methodology: The authors did not specify whether blood samples were collected during an active phase of CSU or during remission. In addition, the sentence regarding treatments (Line 86) is unclear: was there a washout period, or were patients still receiving therapy at the time of sampling? Please clarify this point.

Minor Comments

Line 25: “matching ratio”

Line 170: Spell out “Seventy-three” at the start of the sentence.

Lines 171, 174: Harmonize p-value spacing

Line 178: Add missing space after period: “…(p < 0.001). (Fig 1B)”.

Line 176: Replace pg/ml” by pg/mL throughout.

Lines 243–244: “significantly higher.”

Line 263: Replace that by “by”

Reviewer #2: Review for the manuscript ,,Plasma C5a and serum C5aR levels in patients with chronic spontaneous urticaria: A single-center case-control study,,

In this review the authors tried to analyze plasma C5a and serum C5aR in Vietnamese patients with CSU and also to investigate the association with disease severity.

The results of this study suggest that plasma levels of C5a were significantly elevated in patients with CSU compared to healthy controls and may serve as a predictive biomarker of disease severity.The authors also found that plasma C5a concentrations were significantly correlated with age, sex, family, history of CSU, leukocyte count, erythrocyte sedimentation rate, IgG anti-TPO levels, and PT.

Very interesting subject with great impact od human health.

Comments

Please explain the classification of patients with a UAS7 score of 28 or higher as severe CSU, and those with a UAS7 score below 28 as non-severe CSU.

The authors should indicate the methods and equipment used to determine biochemical parameters.

Reviewer #3: This manuscript addresses an important and timely question concerning the role of complement activation—specifically C5a and soluble C5aR—in chronic spontaneous urticaria (CSU). The topic is relevant, the patient sample is adequate for a single-center study, and the findings add data from an underrepresented population. The study demonstrates a consistent association between elevated plasma C5a levels and CSU severity, contributing to the growing literature on coagulation–complement interactions in urticaria.

However, several substantial issues limit the current quality of the manuscript and preclude acceptance in its present form. These include:

1. “There is a methodological inconsistency regarding the C5a cutoff value. The Abstract and Discussion report 4335 pg/mL, whereas the Results section and Figure 3 indicate 4535 pg/mL. The authors must clarify the correct ROC-derived cutoff and revise the manuscript accordingly.”

2. insufficient characterization of the control group

3. limited explanation and validation of the soluble C5aR assay

4. overinterpretation of cross-sectional data as predictive or mechanistic

5. The manuscript reports significantly higher plasma C5a levels in female CSU patients compared to males; however, this sex difference is not addressed in the Discussion.

6. statistical concerns related to skewness, scaling of effect sizes, and multivariable modeling: No results are shown regarding regression analysis.

**********

6. PLOS authors have the option to publish the peer review history of their article (what does this mean?). If published, this will include your full peer review and any attached files.

If you choose “no”, your identity will remain anonymous but your review may still be made public.

**Do you want your identity to be public for this peer review?**  For information about this choice, including consent withdrawal, please see our Privacy Policy.

Reviewer #1: No

Reviewer #2: No

Reviewer #3: **Yes:**  Young-Min Ye

**********

[NOTE: If reviewer comments were submitted as an attachment file, they will be attached to this email and accessible via the submission site. Please log into your account, locate the manuscript record, and check for the action link "View Attachments". If this link does not appear, there are no attachment files.]

To ensure your figures meet our technical requirements, please review our figure guidelines: https://journals.plos.org/plosone/s/figures

You may also use PLOS’s free figure tool, NAAS, to help you prepare publication quality figures: https://journals.plos.org/plosone/s/figures#loc-tools-for-figure-preparation.

NAAS will assess whether your figures meet our technical requirements by comparing each figure against our figure specifications.

# Author comment: Author response to Decision Letter 1

**DOI:** 10.1371/journal.pone.0351329.r002

22 Jan 2026

Dear the Editor and esteemed reviewers:

We sincerely thank you for your consideration and for rigorously reviewing our manuscript. We realize that your insightful comments significantly help us to improve the scientific rigor and clarity of our study. We have revised the manuscript, accordingly, as outlined below and highlighted within the manuscript.

All authors confirm that this manuscript has not been published elsewhere and is not under consideration by any other journal. All authors declare no conflicts of interest.

Please do not hesitate to let us know if additional information is required. Thank you for your consideration.

Sincerely,

Phuong Thi Minh Pham, MD, PhD

Department of Dermatology, Hanoi Medical University

National Hospital of Dermatology and Venereology, Hanoi, Vietnam

Corresponding author

#Additional Editor Comments:

Thank you for your submission to our Plos One.

I have completed the review process and am pleased to inform that your study is valuable for further consideration.

As you can read the reviews, I would be happy if you appropriately revise the original manuscript.

We sincerely thank the Editor for considering our manuscript for further evaluation. We greatly appreciate the constructive comments provided by the reviewers, which we found immensely valuable. We have carefully addressed all their suggestions and revised the manuscript accordingly to enhance its scientific clarity, methodological transparency, and overall readability. We believe these revisions have strengthened the quality of our work, and we are grateful for the opportunity to improve our study through this process.

Reviewer #1: This manuscript addresses the potential role of complement components C5a and C5aR in the pathophysiology and severity of CSU. The topic is clinically relevant, as CSU remains a heterogeneous and incompletely understood condition. The work provides valuable data from a Southeast Asian population, an underrepresented group in CSU research. The manuscript is clearly written and methodologically sound. The main finding (an increase in plasma C5a levels correlating with CSU severity and a decrease in soluble C5aR) adds incremental information to the literature, particularly since a drug targeting this pathway is currently under development. Compared with previous studies by Zhu et al. (2012), Alizadeh Aghdam et al. (2021), and Bhatia et al. (2024), this manuscript provides a larger cohort, integrating both complement and coagulation pathways in a comprehensive quantitative analysis. Importantly, it is the first to measure soluble C5aR levels and to identify a plasma C5a cutoff predictive of disease severity. However, several major and minor revisions are required before the manuscript can be accepted.

We are grateful for your kind remarks. We greatly appreciate your recognition of our work’s contribution to the literature, particularly in highlighting data from a Southeast Asian population—an underrepresented group in pre-existing CSU research. In the revised manuscript, we have more clearly emphasized this outstanding aspect (lines 269-274) to underline the novelty and importance of our work. We have also carefully addressed the major and minor concerns raised, as detailed in our point‑by‑point responses below. These revisions were made with the aim of improving scientific clarity, methodological transparency, and overall readability.

Major Comments

The elevation of plasma C5a in CSU has been demonstrated in several previous studies, and one has suggested a possible link with disease severity. Please emphasize more clearly the novelty of this study, including the larger cohort size, the use of a standardized scoring system, the definition of a cutoff value, and the measurement of soluble C5aR when citing these references, as this aspect is not sufficiently highlighted in the current version

We sincerely thank the reviewer for drawing attention to the novel contributions of our study. We fully agree that these aspects should be emphasized more clearly. In the revised manuscript, we have explicitly highlighted the unique strengths of our work (lines 269-274). Specifically, our study includes a comparatively large cohort from a Southeast Asian population, which remains underrepresented in CSU research, together with a matched control group for comparison. We have also employed a standardized scoring system (UAS7) to assess disease severity, incorporated ROC analysis to establish a clinically relevant cutoff value for plasma C5a, and, importantly, measured soluble C5aR levels simultaneously for the first time. By underscoring these points, we aim to strengthen the methodological rigor and reaffirm the scientific novelty and robustness of our study.

Line 312–315: Please revise this sentence, as there is an association between C5a levels and CSU severity but no evidence of causality. Therefore, it cannot be concluded that the higher frequency of CSU in women and older patients is caused by increased C5a levels in these groups.

We sincerely apologize for any confusion caused by the original wording and thank the reviewer for highlighting this important point. We fully agree that, while our data demonstrate a statistical association between plasma C5a levels and CSU severity (as assessed by UAS7), this does not establish a causal relationship. Our initial intention was to describe the observation that both C5a levels and CSU severity were higher in female and older patients. However, we recognize that drawing an exposure‑to‑outcome conclusion requires much greater rigor and further investigation. Accordingly, we have revised the sentence to read: “Although there is a statistical association between plasma C5a levels and CSU severity, and both parameters are significantly increased in older and female patients, establishing a causal relationship warrants further studies.” (lines 327-331) We believe this rephrasing improves the clarity and objectivity of our interpretation.

Methodology: The authors did not specify whether blood samples were collected during an active phase of CSU or during remission. In addition, the sentence regarding treatments (Line 86) is unclear: was there a washout period, or were patients still receiving therapy at the time of sampling? Please clarify this point.

We thank the reviewer for highlighting this important point and sincerely apologize for the confusion regarding the description of treatment washout. The original wording was imprecise; our intention was to indicate that participants discontinued relevant medications prior to blood collection. The previous expression “after at least five‑day use” may have caused uncertainty for readers. We have therefore clarified the sentence as: “Peripheral blood samples were obtained from eligible candidates, including CSU patients and healthy controls, after at least five-day discontinuation of second generation H1 antihistamines (sgAH1), one month for immunosuppressant (systemic corticosteroids, cyclosporine A, methotrexate, etc.), and one week for NSAIDs or antibiotics.” (lines 87-90)

Minor Comments

Line 25: “matching ratio”

Line 170: Spell out “Seventy-three” at the start of the sentence.

Lines 171, 174: Harmonize p-value spacing

Line 178: Add missing space after period: “…(p < 0.001). (Fig 1B)”.

Line 176: Replace pg/ml” by pg/mL throughout.

Lines 243–244: “significantly higher.”

Line 263: Replace that by “by”

We sincerely thank the reviewer for carefully identifying these accuracy issues. We apologize for the errors in wording and formatting. In addition to correcting the specific points noted, we have thoroughly reviewed the entire manuscript to ensure consistency and to correct any remaining inaccuracies.

Reviewer #2: Review for the manuscript ,,Plasma C5a and serum C5aR levels in patients with chronic spontaneous urticaria: A single-center case-control study,,

In this review the authors tried to analyze plasma C5a and serum C5aR in Vietnamese patients with CSU and also to investigate the association with disease severity.

The results of this study suggest that plasma levels of C5a were significantly elevated in patients with CSU compared to healthy controls and may serve as a predictive biomarker of disease severity.The authors also found that plasma C5a concentrations were significantly correlated with age, sex, family, history of CSU, leukocyte count, erythrocyte sedimentation rate, IgG anti-TPO levels, and PT.

Very interesting subject with great impact od human health.

Thank you very much for your kind remarks and for acknowledging the significance of our work.

Comments

Please explain the classification of patients with a UAS7 score of 28 or higher as severe CSU, and those with a UAS7 score below 28 as non-severe CSU.

The authors should indicate the methods and equipment used to determine biochemical parameters.

We thank the reviewer for raising this important concern. To clarify, our urticaria clinic and this study employ the internationally accepted Urticaria Activity Score over 7 days (UAS7), which is the gold‑standard tool recommended by the EAACI/GA²LEN/EDF/WAO guidelines for assessing CSU activity. According to these guidelines, CSU severity is classified into four categories: well‑controlled (0–6), mild (7–15), moderate (16–27), and severe (≥28). In our cohort, which included patients who had discontinued relevant medications prior to sampling, approximately 50% of patients had severe CSU and 50% had mild to moderate disease. This distribution indicates a right‑skewed severity profile, with the majority of patients presenting with severe disease. As our study aimed to conduct ROC analysis to identify a predictive cut‑off value, the outcome variable needed to be binary. Therefore, we categorized patients into severe (UAS7 ≥28) and non‑severe (UAS7 <28) groups. This approach is consistent with clinical practice and enhances the robustness of statistical analysis. Furthermore, similar methodology has been adopted in previous studies, such as Lao K. et al. (Clin Exp Allergy, 2024, https://onlinelibrary.wiley.com/doi/10.1111/cea.14531) and Nguyen et al. (Sci Rep, 2025, https://www.nature.com/articles/s41598-025-94841-1). Nevertheless, we acknowledge that further studies with larger proprietary cohorts are warranted to fully substantiate the four‑tier classification of CSU severity.

In addition, we have clarified the methods and equipment used to determine biochemical parameters. Levels of plasma C5a and serum C5aR, the primarily investigated parameters, were measured using commercially available ELISA kits (My BioSource, Inc, San Diego, CA, USA) according to the manufacturer’s instructions. Additional biochemical parameters, which further characterized the cohort, were quantified at the Department of Laboratory Medicine with ISO qualified. These details have been added to the Methods section (lines 104-109) to ensure transparency and reproducibility.

Reviewer #3: This manuscript addresses an important and timely question concerning the role of complement activation—specifically C5a and soluble C5aR—in chronic spontaneous urticaria (CSU). The topic is relevant, the patient sample is adequate for a single-center study, and the findings add data from an underrepresented population. The study demonstrates a consistent association between elevated plasma C5a levels and CSU severity, contributing to the growing literature on coagulation–complement interactions in urticaria.

We greatly appreciate your kind remarks and highlighting important contributions of our study. We are pleased to address the concerns you raised to enhance the scientific clarity of the manuscript.

However, several substantial issues limit the current quality of the manuscript and preclude acceptance in its present form. These include:

1. “There is a methodological inconsistency regarding the C5a cutoff value. The Abstract and Discussion report 4335 pg/mL, whereas the Results section and Figure 3 indicate 4535 pg/mL. The authors must clarify the correct ROC-derived cutoff and revise the manuscript accordingly.”

We sincerely apologize for this typographical error. We have corrected the cut-off value of C5a differentiating severe and non-severe CSU. The accurate value of 4335 pg/mL has been revised to be consistent across the manuscript.

2. insufficient characterization of the control group

We thank the reviewer for raising this valuable observation. We fully agree that comprehensive characterization of the control group is essential to ensure the validity of comparisons between CSU patients and healthy controls. We have acknowledged this limitation in the revised manuscript (lines 348-349) to more clearly frame the interpretation of our findings. Nevertheless, the healthy controls in our study were sex- and age-matched to CSU patients and were carefully screened according to the inclusion criteria described in the Materials and Methods section (lines 87-94), which excluded individuals with prior diseases potentially affecting C5a/C5aR levels or recent use of relevant medications. While the two groups were not fully matched in terms of clinical and laboratory parameters, our approach mitigated several important confounding factors, including age, sex, concomitant medical conditions, and therapies.

3. limited explanation and validation of the soluble C5aR assay

We appreciate the reviewer’s insightful comment regarding the explanation and validation of the soluble C5aR assay. To ensure clarity, we have more explicitly described the assay procedures in the revised Materials and Methods section (lines 113-116) and have attached the original manufacturer’s documentation with this resubmission. The C5a and soluble C5aR assays employed in our study were developed by MyBioSource, a well-established provider of ELISA kits for research purposes. As detailed in the manufacturer’s protocol, soluble C5aR measurement is feasible across various sample types, including blood, urine, cell culture supernatants, and tissue homogenates. In our study, the primary objective was to assess circulating C5aR levels in patients with CSU, and all laboratory procedures—including specimen collection and storage, reagent preparation, assay execution, and data interpretation—were conducted in strict accordance with the manufacturer’s guidelines.

4. overinterpretation of cross-sectional data as predictive or mechanistic

We thank the reviewer for this constructive feedback. We completely agree that findings derived from a cross-sectional study should be interpreted as indicative rather than predictive or mechanistic. Accordingly, we have revised the manuscript (lines 44, 219, 267, 357) to ensure that the conclusions are aligned with the actual robustness of our study. In addition, we have explicitly acknowledged the inherent limitations of cross-sectional evidence and have clearly stated that further longitudinal investigations are warranted (lines 345-346, 352-354).

5. The manuscript reports significantly higher plasma C5a levels in female CSU patients compared to males; however, this sex difference is not addressed in the Discussion.

We thank the reviewer for highlighting this important point. We apologize that the sex-related difference in plasma C5a levels was not clearly addressed in the Discussion. In the revised manuscript (lines 327-331), we have explicitly discussed the significantly higher plasma C5a levels observed in female CSU patients compared to males which can be explained by higher frequency of Type IIb endotype CSU among females.

6. statistical concerns related to skewness, scaling of effect sizes, and multivariable modeling: No results are shown regarding regression analysis.

We thank the reviewer for this thoughtful consideration regarding the statistical analysis. In response, we have conducted additional analyses to rigorously evaluate the robustness of the associations and the applicability of our results. To address the skewness of C5a/C5aR levels and to minimize the influence of effect size scaling, we applied log transformation and standardized the variables by 1 SD. These adjustments allowed for clearer interpretation of the findings within the multivariable model. The revised results are now presented in Table 2, alongside the original scaling of C5a/C5aR

Attachment Submitted filename: R1. Response to Reviewer.docx

# Peer review: Decision Letter 1

**DOI:** 10.1371/journal.pone.0351329.r003

**Authors:** Cheorl-Ho Kim, Cheorl-Ho Kim

4 Mar 2026

<div>PONE-D-25-49336R1

Plasma C5a and serum C5aR levels in patients with chronic spontaneous urticaria: A single-center case-control study

PLOS One

Dear Dr. Pham,

Thank you for submitting your manuscript to PLOS ONE. After careful consideration, we feel that it has merit but does not fully meet PLOS ONE’s publication criteria as it currently stands. Therefore, we invite you to submit a revised version of the manuscript that addresses the points raised during the review process.

Please submit your revised manuscript by Apr 18 2026 11:59PM. If you will need more time than this to complete your revisions, please reply to this message or contact the journal office at plosone@plos.org. When you're ready to submit your revision, log on to https://www.editorialmanager.com/pone/ and select the 'Submissions Needing Revision' folder to locate your manuscript file.

Please include the following items when submitting your revised manuscript:

- A letter that responds to each point raised by the academic editor and reviewer(s). You should upload this letter as a separate file labeled 'Response to Reviewers'.
- A marked-up copy of your manuscript that highlights changes made to the original version. You should upload this as a separate file labeled 'Revised Manuscript with Track Changes'.
- An unmarked version of your revised paper without tracked changes. You should upload this as a separate file labeled 'Manuscript'.

If you would like to make changes to your financial disclosure, please include your updated statement in your cover letter. Guidelines for resubmitting your figure files are available below the reviewer comments at the end of this letter.

If applicable, we recommend that you deposit your laboratory protocols in protocols.io to enhance the reproducibility of your results. Protocols.io assigns your protocol its own identifier (DOI) so that it can be cited independently in the future. For instructions see: https://journals.plos.org/plosone/s/submission-guidelines#loc-laboratory-protocols. Additionally, PLOS ONE offers an option for publishing peer-reviewed Lab Protocol articles, which describe protocols hosted on protocols.io. Read more information on sharing protocols at https://plos.org/protocols?utm_medium=editorial-email&utm_source=authorletters&utm_campaign=protocols.

We look forward to receiving your revised manuscript.

Kind regards,

Cheorl-Ho Kim, Ph.D.

Academic Editor

PLOS One

Journal Requirements:

If the reviewer comments include a recommendation to cite specific previously published works, please review and evaluate these publications to determine whether they are relevant and should be cited. There is no requirement to cite these works unless the editor has indicated otherwise.

Additional Editor Comments:

I have completed a second ground's reviewing because your study can be documented (by editorial point) due to its relevant results in this specific field.

I would be happy if you could carefully re-revise the revised version.

Thanks a lot

Best regards

Cheorl-Ho Kim

[Note: HTML markup is below. Please do not edit.]

Reviewers' comments:

Reviewer's Responses to Questions

**Comments to the Author**

1. If the authors have adequately addressed your comments raised in a previous round of review and you feel that this manuscript is now acceptable for publication, you may indicate that here to bypass the “Comments to the Author” section, enter your conflict of interest statement in the “Confidential to Editor” section, and submit your "Accept" recommendation.

Reviewer #4: All comments have been addressed

Reviewer #5: (No Response)

**********

2. Is the manuscript technically sound, and do the data support the conclusions?

The manuscript must describe a technically sound piece of scientific research with data that supports the conclusions. Experiments must have been conducted rigorously, with appropriate controls, replication, and sample sizes. The conclusions must be drawn appropriately based on the data presented.

Reviewer #4: Yes

Reviewer #5: Partly

**********

3. Has the statistical analysis been performed appropriately and rigorously?

Reviewer #4: Yes

Reviewer #5: Yes

**********

4. Have the authors made all data underlying the findings in their manuscript fully available?

The PLOS Data policy requires authors to make all data underlying the findings described in their manuscript fully available without restriction, with rare exception (please refer to the Data Availability Statement in the manuscript PDF file). The data should be provided as part of the manuscript or its supporting information, or deposited to a public repository. For example, in addition to summary statistics, the data points behind means, medians and variance measures should be available. If there are restrictions on publicly sharing data—e.g. participant privacy or use of data from a third party—those must be specified.

Reviewer #4: Yes

Reviewer #5: Yes

**********

5. Is the manuscript presented in an intelligible fashion and written in standard English?

PLOS ONE does not copyedit accepted manuscripts, so the language in submitted articles must be clear, correct, and unambiguous. Any typographical or grammatical errors should be corrected at revision, so please note any specific errors here.

Reviewer #4: Yes

Reviewer #5: Yes

**********

6. Review Comments to the Author

Please use the space provided to explain your answers to the questions above. You may also include additional comments for the author, including concerns about dual publication, research ethics, or publication ethics. (Please upload your review as an attachment if it exceeds 20,000 characters)

Reviewer #4: Thanks to authors for the revision. In my opionion, these results are helpful for understanding the mechanism of urticaria especially in different populations. I have no additional comments for the manuscript.

Reviewer #5: Dear Authors,

I have carefully reviewed the manuscript entitled “Plasma C5a and serum C5aR levels in patients with chronic spontaneous urticaria: A single-center case-control study.” This study investigates the association between circulating C5a and soluble C5aR levels and disease severity in chronic spontaneous urticaria (CSU), focusing on a Vietnamese cohort.

The topic is timely and relevant. Increasing evidence supports the interplay between complement activation, coagulation pathways, and mast cell activation in CSU pathogenesis. The authors address an important gap, particularly regarding soluble C5aR levels, which have not previously been extensively evaluated in CSU. The relatively large patient sample (n=146), use of standardized disease severity assessment (UAS7), and multivariate modeling represent strengths of the study.

However, several methodological and interpretative issues should be addressed before the manuscript can be considered for publication.

Major comments

1. Study Design and Control Group Size

Although the patient sample is adequate, the control group (n=30) is relatively small compared to the CSU cohort (5:1 matching). This imbalance may affect statistical robustness and ROC curve interpretation. The authors should clarify whether power analysis was performed and how matching was implemented in the statistical analysis (beyond demographic matching).

2. Pre-Analytical Conditions and Medication Washout

The manuscript states that blood sampling was performed after specific washout periods for antihistamines and immunosuppressants. The rationale for the specified washout durations (chosen according to what) should be justified. IS five days enough for the immunosupresant especially systemic corticosteroids?

3. Measurement of “Serum C5aR”

The biological relevance of soluble C5aR requires further clarification:

• What form of C5aR is detected by the ELISA kit (full-length receptor, shed fragment, or splice variant)?

• Is the assay validated for serum measurement?

• The discussion on receptor internalization is speculative and should be clearly presented as hypothesis rather than established mechanism.

Given that membrane-bound C5aR expression on immune cells was not assessed, the conclusions regarding receptor dynamics should be tempered.

4. Statistical Considerations

Several statistical concerns warrant clarification:

• The authors state that linear regression was performed, yet correlation coefficients reported are Spearman’s rho. This should be corrected for clarity.

• ROC analysis reports 100% specificity. This is unusually high and raises concerns about overfitting, particularly given the modest control size. Cross-validation or bootstrapping would strengthen this analysis.

• Multiple testing correction was not mentioned despite numerous correlation analyses.

5. Interpretation of Coagulation–Complement Interaction

While the mechanistic discussion linking extrinsic coagulation activation to complement activation is comprehensive, it is largely inferential in this study. Since direct markers such as F1+2, TAT, or FVIIa were not measured, the authors should avoid overextending mechanistic conclusions beyond the observed associations.

Minor Comments

1. The ellipsis (“…”) at the end of the sentence describing C5aR expression appears inappropriate in an academic manuscript. Please revise the sentence to remove the ellipsis or replace it with a precise expression (e.g., “among others”) if additional cell types are intended.

2. The description of medication management prior to blood sampling is unclear and requires clarification. The sentence currently states that samples were obtained “after at least five-day use of generation H1 antihistamines,” which is ambiguous and grammatically incorrect. It is not clear whether blood samples were collected after a defined washout period following discontinuation of these medications or after a minimum duration of use. Please clarify the medication protocol, explicitly specifying whether a washout period was applied, and indicate the exact duration of discontinuation for second-generation H1 antihistamines, immunosuppressants (particularly systemic corticosteroids), NSAIDs, and antibiotics. Given that these agents may significantly influence complement and inflammatory markers, this methodological detail is essential for accurate interpretation of the results.

3. Peripheral blood samples were obtained from eligible candidates after at least five-day use of generation H1 antihistamines (sgAH1),..Here, please insert ‘second’ before ‘generation’.

4. The manuscript does not specify whether any patients were receiving omalizumab therapy at the time of enrollment or blood sampling. Given the potential immunomodulatory effects of omalizumab and its impact on disease activity and inflammatory pathways, this information is essential. Please clarify whether patients on omalizumab were included, excluded, or analyzed separately.

5. I suggest moving the (Patients diagnosed or suspected of having chronic inducible urticaria (CIndU) were excluded from the study) sentence to the exclusion criteria section to improve the clarity and structure of the Methods.

6. There are several grammatical inconsistencies and typographical errors (e.g., “maching ratio,” “plasm C5a,” inconsistent spacing, and tense issues). Professional language editing is recommended.

7. Units should be standardized (pg/mL rather than pg/ml).

8. The cutoff value for C5a is inconsistently reported (4335 pg/mL in the abstract vs. 4535 pg/mL in the Results section). This discrepancy must be corrected.

9. Clarify whether C5a measurements were performed in duplicate and report intra-/inter-assay coefficients of variation.

10. The term “predictive biomarker” should be used cautiously in cross-sectional case-control studies; “associated biomarker” may be more appropriate.

11. The discussion could benefit from briefly addressing how these findings might integrate with current biologic therapies (e.g., anti-IgE therapy) in CSU.

Overall Recommendation

This study contributes valuable data regarding complement activation in CSU and introduces novel information on circulating C5aR levels. However, important methodological clarifications, statistical refinement, and moderation of mechanistic interpretations are required.

I recommend major revision prior to reconsideration.

Regards,

**********

7. PLOS authors have the option to publish the peer review history of their article (what does this mean?). If published, this will include your full peer review and any attached files.

If you choose “no”, your identity will remain anonymous but your review may still be made public.

**Do you want your identity to be public for this peer review?**  For information about this choice, including consent withdrawal, please see our Privacy Policy.

Reviewer #4: No

Reviewer #5: **Yes:**  Semra Demir

**********

[NOTE: If reviewer comments were submitted as an attachment file, they will be attached to this email and accessible via the submission site. Please log into your account, locate the manuscript record, and check for the action link "View Attachments". If this link does not appear, there are no attachment files.]

To ensure your figures meet our technical requirements, please review our figure guidelines: https://journals.plos.org/plosone/s/figures

You may also use PLOS’s free figure tool, NAAS, to help you prepare publication quality figures: https://journals.plos.org/plosone/s/figures#loc-tools-for-figure-preparation.

NAAS will assess whether your figures meet our technical requirements by comparing each figure against our figure specifications.

Attachment Submitted filename: comments for authors.docx

# Author comment: Author response to Decision Letter 2

**DOI:** 10.1371/journal.pone.0351329.r004

9 Apr 2026

RESPONSE TO REVIEWERS

Article title: Plasma C5a and serum C5aR levels in patients with chronic spontaneous urticaria: A single-center case-control study

Short title: C5a and C5aR in chronic spontaneous urticaria

Authors: Thao T. Pham, MD1,2, Minh N. Vu, MD, PhD1,2, My H. Le, MD, PhD2, Doanh H. Le, MD, PhD1,2, Thuong V. Nguyen, MD, PhD1,2, Phuong T.M. Pham, MD, PhD2

Affiliations:

1 Department of Dermatology, Hanoi Medical University, Hanoi, Vietnam

2 National Hospital of Dermatology and Venereology, Hanoi, Vietnam

Corresponding author: Phuong T.M. Pham, MD, PhD; National Hospital of Dermatology and Venereology, Hanoi, Vietnam; Email: phuongphamdv@gmail.com

Additional Editor Comments:

I have completed a second ground's reviewing because your study can be documented (by editorial point) due to its relevant results in this specific field.

I would be happy if you could carefully re-revise the revised version.

Thanks a lot

Best regards

Cheorl-Ho Kim

We sincerely thank Dr. Kim – the handing editor – for kind remarks and for dedicatedly reviewing our manuscript and grateful to address all concerns raised by esteemed reviewers, as outlined point-by-point below:

6. Review Comments to the Author

Reviewer #4: Thanks to authors for the revision. In my opionion, these results are helpful for understanding the mechanism of urticaria especially in different populations. I have no additional comments for the manuscript.

We greatly appreciate the reviewer’s positive remarks and recognition of the value of our study. We are pleased that the findings made contributions to the evolving understanding of the mechanisms of urticaria across different populations.

Reviewer #5: Dear Authors,

I have carefully reviewed the manuscript entitled “Plasma C5a and serum C5aR levels in patients with chronic spontaneous urticaria: A single-center case-control study.” This study investigates the association between circulating C5a and soluble C5aR levels and disease severity in chronic spontaneous urticaria (CSU), focusing on a Vietnamese cohort.

The topic is timely and relevant. Increasing evidence supports the interplay between complement activation, coagulation pathways, and mast cell activation in CSU pathogenesis. The authors address an important gap, particularly regarding soluble C5aR levels, which have not previously been extensively evaluated in CSU. The relatively large patient sample (n=146), use of standardized disease severity assessment (UAS7), and multivariate modeling represent strengths of the study.

However, several methodological and interpretative issues should be addressed before the manuscript can be considered for publication.

We thank the reviewer for the objective and constructive comments. We acknowledge that several methodological and interpretative issues required clarification, and we have carefully revised the manuscript to address these concerns. Specifically, we have provided additional methodological details, refined the statistical analyses, and moderated the mechanistic interpretations. We believe these revisions have substantially improved the clarity, rigor, and readability of our work, and we hope the revised version meets the reviewer’s expectations.

Major comments

1. Study Design and Control Group Size

Although the patient sample is adequate, the control group (n=30) is relatively small compared to the CSU cohort (5:1 matching). This imbalance may affect statistical robustness and ROC curve interpretation. The authors should clarify whether power analysis was performed and how matching was implemented in the statistical analysis (beyond demographic matching).

Thank you very much for pointing out this important consideration. We sincerely apologize for the lack of explanation regarding the matching procedure. In the revised manuscript (lines 85-86), we have clarified that, due to the limited number of healthy controls, CSU patients were grouped in sets of five with the closest age and each group was matched to one healthy donor of similar age. We agree that the 5:1 matching ratio may influence statistical robustness. Accordingly, we have explicitly acknowledged this limitation (lines 364-366) and performed additional analyses to strengthen our findings, including bootstrapping procedures for ROC curve interpretation and multiple testing correction for correlation analyses. These revisions have substantially improved the methodological clarity and scientific rigor of our work.

2. Pre-Analytical Conditions and Medication Washout

The manuscript states that blood sampling was performed after specific washout periods for antihistamines and immunosuppressants. The rationale for the specified washout durations (chosen according to what) should be justified. IS five days enough for the immunosuppresant especially systemic corticosteroids?

We thank the reviewer for this insightful observation and are pleased to elaborate on the rationale for the specified washout periods. Based on pharmacokinetics, approximately five half-lives are required for a drug to be considered cleared. In our cohort, patients were treated with bilastine or rupatadine, with half-lives of 14.5 and 5.9 hours, respectively, and thus a 5‑day washout was sufficient. For immunosuppressants, including systemic corticosteroids, cyclosporine, and methotrexate, a washout period of one month (30 days) was implemented to ensure clearance across systemic agents. These details have been clarified in the revised manuscript (lines 89-94).

3. Measurement of “Serum C5aR”

The biological relevance of soluble C5aR requires further clarification:

• What form of C5aR is detected by the ELISA kit (full-length receptor, shed fragment, or splice variant)?

• Is the assay validated for serum measurement?

• The discussion on receptor internalization is speculative and should be clearly presented as hypothesis rather than established mechanism.

Given that membrane-bound C5aR expression on immune cells was not assessed, the conclusions regarding receptor dynamics should be tempered.

We appreciate the reviewer’s insightful comment regarding the explanation and validation of the soluble C5aR assay. To ensure clarity, we have more explicitly described the assay procedures in the revised Materials and Methods section (lines 114-122) and attached the manufacturer’s documentation with this resubmission. The ELISA kits for C5a and soluble C5aR (MyBioSource) are validated for use with multiple sample types, including serum, plasma, urine, cell culture supernatants, and tissue homogenates. Since serum was used in our study, the detected C5aR represents the circulating soluble receptor, as specified by the manufacturer. All laboratory procedures—including specimen collection and storage, reagent preparation, assay execution, and data interpretation—were conducted in strict accordance with the manufacturer’s guidelines. Regarding receptor internalization, we fully agree that this remains speculative. We have revised the Discussion (lines 295-301) to present this point as a hypothesis rather than an established mechanism, and we have tempered our conclusions accordingly.

4. Statistical Considerations

Several statistical concerns warrant clarification:

• The authors state that linear regression was performed, yet correlation coefficients reported are Spearman’s rho. This should be corrected for clarity.

• ROC analysis reports 100% specificity. This is unusually high and raises concerns about overfitting, particularly given the modest control size. Cross-validation or bootstrapping would strengthen this analysis.

• Multiple testing correction was not mentioned despite numerous correlation analyses.

We greatly appreciate the reviewer’s insightful and constructive comments related to our statistical analyses. We agree that these considerations are critical to strengthening the robustness of our findings. We are pleased to address as follows:

- Linear regression vs. correlation coefficients: We apologize for the inconsistency in terminology. Since the variables analyzed were non-normally distributed, we employed Spearman’s correlation to assess associations between C5a/C5aR levels and clinical parameters. The manuscript has been revised accordingly to ensure consistent and accurate phrasing.

- ROC analysis specificity: We acknowledge the reviewer’s concern regarding the unusually high specificity observed in our ROC analysis, particularly given the modest control size. To address this, we performed bootstrapping procedures to evaluate the robustness of the ROC estimates. The bootstrapped results confirmed high specificity, but with confidence intervals reflecting the limited control sample size. It is important to note that the specificity of 100% does not imply 100% certainty. It only reflects that zero false positives occurred in our sample. These details have been incorporated into the revised Methods and Results (lines 149-151 and 234-235), and the Discussion (lines 366-367 and 371-374) now explicitly acknowledges the potential for overfitting and the need for validation in larger, independent cohorts.

- Multiple testing correction for correlation analyses: Multiple testing correction: We appreciate the reviewer’s emphasis on controlling for type I error in the context of multiple correlation analyses. In the revised manuscript, we have specified that the Benjamini–Hochberg procedure was applied to adjust p-values across all correlation tests. Corrected p-values are now reported in Table S2, and the Methods section has been updated accordingly (lines 151-154).

We believe these revisions substantially improve the methodological clarity, transparency, and statistical rigor of our study.

5. Interpretation of Coagulation–Complement Interaction

While the mechanistic discussion linking extrinsic coagulation activation to complement activation is comprehensive, it is largely inferential in this study. Since direct markers such as F1+2, TAT, or FVIIa were not measured, the authors should avoid overextending mechanistic conclusions beyond the observed associations.

We appreciate the reviewer’s insightful comment regarding the interpretation of coagulation–complement interactions. We agree that our mechanistic discussion was largely inferential, as direct markers such as F1+2, TAT, or FVIIa were not measured in this study. In response, we have revised the Discussion to temper our language, clearly distinguishing observed associations from mechanistic speculation (Paragraph lines 309-337 where highlighted). We now emphasize that while our findings are consistent with the hypothesis of extrinsic coagulation activation contributing to complement activation, definitive mechanistic conclusions cannot be drawn without direct measurement of coagulation activation markers. We believe these revisions provide a more balanced and appropriately cautious interpretation of our results.

Minor Comments

1. The ellipsis (“…”) at the end of the sentence describing C5aR expression appears inappropriate in an academic manuscript. Please revise the sentence to remove the ellipsis or replace it with a precise expression (e.g., “among others”) if additional cell types are intended.

We thank the reviewer for pointing out the inappropriate use of an ellipsis in the description of C5aR expression. We have revised the sentence to remove the ellipsis and replaced it with a precise expression (“among other cell types”) to ensure clarity and adherence to academic style (line 75).

2. The description of medication management prior to blood sampling is unclear and requires clarification. The sentence currently states that samples were obtained “after at least five-day use of generation H1 antihistamines,” which is ambiguous and grammatically incorrect. It is not clear whether blood samples were collected after a defined washout period following discontinuation of these medications or after a minimum duration of use. Please clarify the medication protocol, explicitly specifying whether a washout period was applied, and indicate the exact duration of discontinuation for second-generation H1 antihistamines, immunosuppressants (particularly systemic corticosteroids), NSAIDs, and antibiotics. Given that these agents may significantly influence complement and inflammatory markers, this methodological detail is essential for accurate interpretation of the results.

We sincerely thank the reviewer for highlighting the need for clarification regarding medication management prior to blood sampling. In the revised manuscript, we have specified the exact washout periods applied. “Peripheral blood samples were obtained from eligible candidates, including CSU patients and healthy controls, after at least five-day discontinuation of second generation H1 antihistamines (sgAH1), one month for immunosuppressants (systemic corticosteroids, cyclosporine A, methotrexate, etc.), and one week for NSAIDs or antibiotics” (lines 89-94).

3. Peripheral blood samples were obtained from eligible candidates after at least five-day use of generation H1 antihistamines (sgAH1),..Here, please insert ‘second’ before ‘generation’.

We thank the reviewer for drawing attention to this typographical omission. The sentence has been corrected to read “second-generation H1 antihistamines (sgAH1)” in the revised manuscript. We regret the oversight and have ensured that the terminology is now consistent and accurate throughout the text.

4. The manuscript does not specify whether any patients were receiving omalizumab therapy at the time of enrollment or blood sampling. Given the potential immunomodulatory effects of omalizumab and its impact on disease activity and inflammatory pathways, this information is essential. Please clarify whether patients on omalizumab were included, excluded, or analyzed separately.

We sincerely thank the reviewer for raising this important point regarding omalizumab therapy. Omalizumab, although increasingly utilized in the management of CSU, has not been widely prescribed in our urticaria clinic due to its high financial cost and lack of insurance coverage. In practice, only a very small proportion of patients (approximately 1%) are able to access this biologic agent. Moreover, given the long half-life of omalizumab (approximately 26 days), recruitment of patients with recent exposure was not feasible. During the study period, no CSU patients with a history of omalizumab use met the eligibility criteria. We have added this clarification to the Discussion (lines 367-369, 371-374), noting that future studies with larger and more diverse patient populations—including those receiving biologic therapies—will be necessary to fully elucidate this important research question.

5. I suggest moving the (Patients diagnosed or suspected of having chronic inducible urticaria (CIndU) were excluded from the study) sentence to the exclusion criteria section to improve the clarity and structure of the Methods.

Thank you very much for this appropriate suggestion. The sentence you referred to has been relocated in the Study design and population to improve coherence and structure of the Methods. (lines 88-89).

6. There are several grammatical inconsistencies and typographical errors (e.g., “maching ratio,” “plasm C5a,” inconsistent spacing, and tense issues). Professional language editing is recommended.

7. Units should be standardized (pg/mL rather than pg/ml).

8. The cutoff value for C5a is inconsistently reported (4335 pg/mL in the abstract vs. 4535 pg/mL in the Results section). This discrepancy must be corrected.

We sincerely apologize for the typographical and grammatical inconsistencies identified in the manuscript. In response, we have conducted a thorough review and implemented comprehensive language editing to ensure clarity, accuracy, and consistency throughout the text. These revisions have been carefully applied to enhance the overall quality and precision of the manuscript.

9. Clarify whether C5a measurements were performed in duplicate and report intra-/inter-assay coefficients of variation.

We extremely appreciate this reviewer’s comment on the laboratory procedures in our study. To ensure the validity of the C5a and C5aR measurements, we confirm that all C5a and C5aR measurements were performed in duplicate

Attachment Submitted filename: R2. Response to Reviewer.docx

# Peer review: Decision Letter 2

**DOI:** 10.1371/journal.pone.0351329.r005

**Authors:** Cheorl-Ho Kim, Cheorl-Ho Kim, Cheorl-Ho Kim

26 May 2026

Plasma C5a and serum C5aR levels in patients with chronic spontaneous urticaria: A single-center case-control study

PONE-D-25-49336R2

Dear Dr. Pham,

We’re pleased to inform you that your manuscript has been judged scientifically suitable for publication and will be formally accepted for publication once it meets all outstanding technical requirements.

Within one week, you’ll receive an e-mail detailing the required amendments. When these have been addressed, you’ll receive a formal acceptance letter and your manuscript will be scheduled for publication.

An invoice will be generated when your article is formally accepted. Please note, if your institution has a publishing partnership with PLOS and your article meets the relevant criteria, all or part of your publication costs will be covered. Please make sure your user information is up-to-date by logging into Editorial Manager at Editorial Manager® and clicking the ‘Update My Information' link at the top of the page. For questions related to billing, please contact billing support.

If your institution or institutions have a press office, please notify them about your upcoming paper to help maximize its impact. If they’ll be preparing press materials, please inform our press team as soon as possible -- no later than 48 hours after receiving the formal acceptance. Your manuscript will remain under strict press embargo until 2 pm Eastern Time on the date of publication. For more information, please contact onepress@plos.org.

Kind regards,

Cheorl-Ho Kim, Ph.D.

Academic Editor

PLOS One

Additional Editor Comments (optional):

Dear Dr Phuong T M Pham

Thank you for your patience in waiting for my decision.

I am pleased to inform that you revision is now acceptable for publication in Plos One.

Thank you

Sincerely

Cheorl-Ho Kim PhD Prof

Biological Science

Sungkyunkwan University

Korea

Reviewers' comments:

Reviewer's Responses to Questions

**Comments to the Author**

1. If the authors have adequately addressed your comments raised in a previous round of review and you feel that this manuscript is now acceptable for publication, you may indicate that here to bypass the “Comments to the Author” section, enter your conflict of interest statement in the “Confidential to Editor” section, and submit your "Accept" recommendation.

Reviewer #5: All comments have been addressed

**********

2. Is the manuscript technically sound, and do the data support the conclusions?

The manuscript must describe a technically sound piece of scientific research with data that supports the conclusions. Experiments must have been conducted rigorously, with appropriate controls, replication, and sample sizes. The conclusions must be drawn appropriately based on the data presented.

Reviewer #5: Yes

**********

3. Has the statistical analysis been performed appropriately and rigorously?

Reviewer #5: Yes

**********

4. Have the authors made all data underlying the findings in their manuscript fully available?

The PLOS Data policy requires authors to make all data underlying the findings described in their manuscript fully available without restriction, with rare exception (please refer to the Data Availability Statement in the manuscript PDF file). The data should be provided as part of the manuscript or its supporting information, or deposited to a public repository. For example, in addition to summary statistics, the data points behind means, medians and variance measures should be available. If there are restrictions on publicly sharing data—e.g. participant privacy or use of data from a third party—those must be specified.

Reviewer #5: Yes

**********

5. Is the manuscript presented in an intelligible fashion and written in standard English?

PLOS ONE does not copyedit accepted manuscripts, so the language in submitted articles must be clear, correct, and unambiguous. Any typographical or grammatical errors should be corrected at revision, so please note any specific errors here.

Reviewer #5: Yes

**********

6. Review Comments to the Author

Please use the space provided to explain your answers to the questions above. You may also include additional comments for the author, including concerns about dual publication, research ethics, or publication ethics. (Please upload your review as an attachment if it exceeds 20,000 characters)

Reviewer #5: The manuscript is well written and statistically appropriate. The authors have adequately addressed my comments. I have no further comments.

**********

7. PLOS authors have the option to publish the peer review history of their article (what does this mean?). If published, this will include your full peer review and any attached files.

If you choose “no”, your identity will remain anonymous but your review may still be made public.

**Do you want your identity to be public for this peer review?**  For information about this choice, including consent withdrawal, please see our Privacy Policy.

Reviewer #5: No

**********

# Editor report: Acceptance letter

**DOI:** 10.1371/journal.pone.0351329.r006

**Authors:** Cheorl-Ho Kim, Cheorl-Ho Kim, Cheorl-Ho Kim

PONE-D-25-49336R2

PLOS One

Dear Dr. Pham,

I'm pleased to inform you that your manuscript has been deemed suitable for publication in PLOS One. Congratulations! Your manuscript is now being handed over to our production team.

At this stage, our production department will prepare your paper for publication. This includes ensuring the following:

* All references, tables, and figures are properly cited

* All relevant supporting information is included in the manuscript submission,

* There are no issues that prevent the paper from being properly typeset

You will receive further instructions from the production team, including instructions on how to review your proof when it is ready. Please keep in mind that we are working through a large volume of accepted articles, so please give us a few days to review your paper and let you know the next and final steps.

Lastly, if your institution or institutions have a press office, please let them know about your upcoming paper now to help maximize its impact. If they'll be preparing press materials, please inform our press team within the next 48 hours. Your manuscript will remain under strict press embargo until 2 pm Eastern Time on the date of publication. For more information, please contact onepress@plos.org.

You will receive an invoice from PLOS for your publication fee after your manuscript has reached the completed accept phase. If you receive an email requesting payment before acceptance or for any other service, this may be a phishing scheme. Learn how to identify phishing emails and protect your accounts at https://explore.plos.org/phishing.

If we can help with anything else, please email us at customercare@plos.org.

Thank you for submitting your work to PLOS ONE and supporting open access.

Kind regards,

PLOS ONE Editorial Office Staff

on behalf of

Professor Cheorl-Ho Kim

Academic Editor

PLOS One
